# A 3-Biomarker 2-Point-Based Risk Stratification Strategy in Acute Heart Failure

**DOI:** 10.3389/fphys.2021.708890

**Published:** 2021-10-22

**Authors:** Jesús Álvarez-García, Álvaro García-Osuna, Miquel Vives-Borrás, Andreu Ferrero-Gregori, Manuel Martínez-Sellés, Rafael Vázquez, José R. González-Juanatey, Miguel Rivera, Javier Segovia, Domingo Pascual-Figal, Ramón Bover, Ramón Bascompte, Juan Delgado, Andrés Grau Sepúlveda, Alfredo Bardají, Félix Pérez-Villa, José Luis Zamorano, Marisa Crespo-Leiro, Pedro Luis Sánchez, Jordi Ordoñez-Llanos, Juan Cinca

**Affiliations:** ^1^Cardiology Department, Hospital de la Santa Creu i Sant Pau, IIb-SantPau, Centro de Investigación en Red en Enfermedades Cardiovasculares (CIBERCV), Barcelona, Spain; ^2^Cardiology Department, Hospital Ramón y Cajal, Centro de Investigación en Red en Enfermedades Cardiovasculares (CIBERCV), Madrid, Spain; ^3^Biochemistry Department, Hospital de la Santa Creu i Sant Pau, IIb-SantPau, Barcelona, Spain; ^4^Cardiology Department, Hospital Universitario Gregorio Marañón, Centro de Investigación en Red en Enfermedades Cardiovasculares (CIBERCV), Madrid, Spain; ^5^Cardiology Department, Hospital Puerta del Mar, Centro de Investigación en Red en Enfermedades Cardiovasculares (CIBERCV), Cádiz, Spain; ^6^Cardiology Department, Hospital Clínico, Centro de Investigación en Red en Enfermedades Cardiovasculares (CIBERCV), Santiago de Compostela, Spain; ^7^Cardiology Department, Hospital La Fe, Centro de Investigación en Red en Enfermedades Cardiovasculares (CIBERCV), Valencia, Spain; ^8^Cardiology Department, Hospital Puerta de Hierro-Majadahonda, Centro de Investigación en Red en Enfermedades Cardiovasculares (CIBERCV), Madrid, Spain; ^9^Cardiology Department, Hospital Virgen de la Arrixaca, Centro de Investigación en Red en Enfermedades Cardiovasculares (CIBERCV), Murcia, Spain; ^10^Cardiology Department, Hospital Clínico San Carlos, Centro de Investigación en Red en Enfermedades Cardiovasculares (CIBERCV), Madrid, Spain; ^11^Cardiology Department, Hospital Arnau de Vilanova, Centro de Investigación en Red en Enfermedades Cardiovasculares (CIBERCV), Lleida, Spain; ^12^Cardiology Department, Hospital 12 de Octubre, Centro de Investigación en Red en Enfermedades Cardiovasculares (CIBERCV), Madrid, Spain; ^13^Cardiology Department, Hospital Universitario Son Espases, Centro de Investigación en Red en Enfermedades Cardiovasculares (CIBERCV), Palma de Mallorca, Spain; ^14^Cardiology Department, Hospital Juan XXIII, Centro de Investigación en Red en Enfermedades Cardiovasculares (CIBERCV), Tarragona, Spain; ^15^Cardiology Department, Hospital Clinic, Centro de Investigación en Red en Enfermedades Cardiovasculares (CIBERCV), Barcelona, Spain; ^16^Cardiology Department, Hospital Universitario A Coruna, Centro de Investigación en Red en Enfermedades Cardiovasculares (CIBERCV), A Coruna, Spain; ^17^Cardiology Department, Hospital Clínico Universitario, Centro de Investigación en Red en Enfermedades Cardiovasculares (CIBERCV), Salamanca, Spain

**Keywords:** biomarker (BM), panel (C33), acute heart failure (AHF), risk stratification, prognosis

## Abstract

**Introduction and Objectives:** Most multi-biomarker strategies in acute heart failure (HF) have only measured biomarkers in a single-point time. This study aimed to evaluate the prognostic yielding of NT-proBNP, hsTnT, Cys-C, hs-CRP, GDF15, and GAL-3 in HF patients both at admission and discharge.

**Methods:** We included 830 patients enrolled consecutively in a prospective multicenter registry. Primary outcome was 12-month mortality. The gain in the C-index, calibration, net reclassification improvement (NRI), and integrated discrimination improvement (IDI) was calculated after adding each individual biomarker value or their combination on top of the best clinical model developed in this study (C-index 0.752, 0.715–0.789) and also on top of 4 currently used scores (MAGGIC, GWTG-HF, Redin-SCORE, BCN-bioHF).

**Results:** After 12-month, death occurred in 154 (18.5%) cases. On top of the best clinical model, the addition of NT-proBNP, hs-CRP, and GDF-15 above the respective cutoff point at admission and discharge and their delta during compensation improved the C-index to 0.782 (0.747–0.817), IDI by 5% (*p* < 0.001), and NRI by 57% (*p* < 0.001) for 12-month mortality. A 4-risk grading categories for 12-month mortality (11.7, 19.2, 26.7, and 39.4%, respectively; *p* < 0.001) were obtained using combination of these biomarkers.

**Conclusion:** A model including NT-proBNP, hs-CRP, and GDF-15 measured at admission and discharge afforded a mortality risk prediction greater than our clinical model and also better than the most currently used scores. In addition, this 3-biomarker panel defined 4-risk categories for 12-month mortality.

## Introduction

The prognostic stratification of patients with acute heart failure (HF) is essential to establish an appropriate personalized follow-up plan. Cardiac biomarkers have improved the predictive models of HF patients beyond the already well-established clinical risk predictors such as functional class or physical examination (Levy et al., 2006).

More than 10 years ago, Braunwald provided a comprehensive review of the biomarkers related to the different pathophysiological substrates involved in HF (Braunwald, 2008), and remarked the need to identify the biomarkers with independent predictive value in large prospective cohorts of patients. Until now, a substantial number of studies have assessed the prognostic capacity of panels of 3 or more biomarkers in acute HF (Ishii et al., 2002; van Kimmenade et al., 2006; Januzzi et al., 2007; Rehman et al., 2008; Manzano-Fernández et al., 2009, 2011; Niizeki et al., 2009; Zairis et al., 2010; Pascual-Figal et al., 2011; Shah et al., 2012; Bjurman et al., 2013; Lassus et al., 2013; Lok et al., 2013; Richter et al., 2013; Srinivas et al., 2014; Demissei et al., 2016, 2017a,b; Herrero-Puente et al., 2017; Tromp et al., 2017), but only in few of them the biomarkers were analyzed at both hospital admission and discharge (Demissei et al., 2016, 2017a,b). A single-point measurement would not allow to evaluate the width of the pathophysiological changes occurring during the clinical compensation and, moreover, might limit the predictive capacity of the biomarkers. Although the predictive capacity of the single-point measurement can be improved by increasing the number of the biomarkers in the panel, it is theoretically possible that a substantial improvement could be alternatively attained using only few of them, but measured at both admission and discharge. The prognostic yielding of sequential measurements of a single biomarker (van Vark et al., 2017) or a series of them (Demissei et al., 2017a,b) in patients with HF was evaluated in *post hoc* analysis of clinical trials. However, the data derived from clinical trials might not entirely reflect the daily real practice.

Therefore, we aimed to analyze the performance of a multi-biomarker panel covering distinct pathophysiological axes in HF measured both at admission and at hospital discharge, in a nationwide cohort of patients with acute HF (REDINSCOR II registry). We have selected the N-terminal pro-B-type natriuretic peptide (NT-proBNP) as marker of neurohormonal activation and myocyte stretch, high-sensitive T troponin (hs-TnT) linked to myocyte injury, cystatin-C (Cys-C) as indicative of renal dysfunction, growth differentiation factor 15 (GDF-15) and galectin-3 (GAL-3) as markers of matrix remodeling, and high sensitive C reactive protein (hs-CRP) as marker of inflammation.

## Materials and Methods

### Study Population

This is a subanalysis including 830 patients discharged alive with available biomarker data both at admission and discharge from the REDINSCOR II study. This is a multicenter, prospective nationwide registry, which enrolled consecutively patients from 18 secondary and tertiary hospitals since October 2013 to December 2014. Inclusion criteria were: (i) age older than 18 years; (ii) acute HF as the main cause for admission; and (iii) hospitalization ≥ 24 h in the Cardiology Department. Exclusion criteria were: (i) HF episode secondary to ST-segment elevation acute coronary syndrome; (ii) end-stage disease with a life expectancy < 1 year; and (iii) any condition that would prevent an appropriate follow-up. HF was diagnosed in accordance with current HF guidelines (McMurray et al., 2012). The study complies with the Declaration of Helsinki and the protocol was approved by the Ethics Committees of each participating center. All patients gave written informed consent.

### Study Variables

Data were collected using specifically designed web forms and quality controls were done monthly. The following clinical variables were gathered at study inclusion and before discharge: demographic and previous clinical data, case history and physical examination, chest x-rays, ECG, echocardiography, laboratory blood tests, and pharmacological and non-pharmacological treatment (Appendix 1). Standard criteria were used to define the clinical variables. Left ventricular ejection fraction (LVEF) was categorized according to the recent HF European guidelines (Ponikowski et al., 2016).

### Biomarker Panel

Blood samples were obtained by venipuncture within the first 24 h of admission and thereafter at hospital discharge. The samples were centrifuged at 2,500 g for 15 min. Serum and plasma aliquots of 0.5 mL were separated and frozen at –80°C until analysis; all samples of the same individual were processed in the same batch. Biomarker concentrations were measured at a core laboratory (Biochemistry Department, Hospital de la Santa Creu i Sant Pau, Barcelona, Spain). We measured the serum levels of NT-proBNP, hs-cTnT, and GDF-15 by electrochemiluminescence immunoassays, and cystatin-C and hs-CRP by particle-enhanced turbidimetric immunoassays using reagents from Roche Diagnostics (Basel, Switzerland). Galectin-3 was also measured in serum using an enzyme-linked fluorescent immunoassay (BioMérieux, Marcy-l’Étoile, France). The imprecision of all assays was similar or even lower than that reported by manufacturers.

### Follow-Up and Outcomes

In addition to the specific clinical follow-up needed by the patient, the vital status was also checked at 12 months after discharge. We used either telephone interviews or clinical records of hospitals, primary care, or institutional death registries. The primary outcome was all-cause mortality at 12-month after discharge. The secondary outcomes were cardiovascular mortality, HF mortality, and readmission for HF at the same time period. The reported events were reviewed by an *ad hoc* committee.

### Statistical Analysis

Continuous variables are expressed as mean (standard deviation) or as median (interquartile range) whenever appropriate. Differences in continuous variables were tested by the analysis of variance (ANOVA), Student’s *t*-test, or Wilcoxon signed rank test for independent samples. Categorical variables were presented as frequency and percentage. Differences in the categorical variables were assessed by the *χ*^2^ test or by Fisher’s exact test. A two-sided *p*-value < 0.05 was considered statistically significant. Missing data were imputed using the “MICE” package in R (Multivariate Imputation by Chained Equations) whenever necessary (*m* = 1). All the analyses were performed using R (v. 3.2) and STATA (v. 13.1).

Firstly, we developed the best clinical model to predict the occurrence of the primary endpoint using a multivariable Cox regression analysis. Clinical meaningful variables and those showing a *p*-value < 0.1 in the univariate analysis were thereafter included in the multivariate model. A backward stepwise method was used to identify independent predictors with a *p*-value < 0.05 as inclusion or deletion criteria. This model was finally composed of variables at admission (number of HF episodes during the last year, previous stroke, systolic blood pressure, presence of right HF signs, significant mitral valve regurgitation, hyponatremia, and body mass index), and variables at discharge (persisting HF signs, heart rate, left bundle branch block, eGFR < 60 ml/min/1.73 m^2^, and length of hospital stay). The discriminative ability of the model for all-cause mortality at 12-month after discharge assessed by the C-statistic index was 0.752 (95% CI 0.715–0.789).

On top of this clinical model, we then analyzed sequentially the added prognostic value of each individual biomarker and their combinations using the gain in the C-index, calibration (Grønnesby and Borgan, Brier score, Akaike and Bayesian criteria), integrated discrimination improvement (IDI), and net reclassification improvement (NRI). Moreover, we also analyzed the added prognostic value of each individual biomarker and their combinations on top of other well-validated clinical scores usually used in clinical practice as the MAGGIC (Pocock et al., 2013), GWTG-HF (Peterson et al., 2010), Redin-SCORE (Álvarez-García et al., 2015), and BCN bio-HF (Lupón et al., 2014). The ROC curve analysis was used to determine the optimal biomarker cut-off value to predict the primary endpoint employing the Youden criteria.

## Results

### Clinical Characteristics of the Study Population

As shown in Table 1, most patients were elderly, male, Caucasian, had a previous history of HF (60%), and a high Charlson comorbidity index. According to LVEF at admission, 263 patients (32%) were classified as HFrEF, 207 (25%) as HFmrEF and 360 (43%) as HFpEF.

**TABLE 1:** Baseline characteristics of the study population.

	**Total (*N* = 830 patients)**
Age, years, median (IQR)	75 (65–82)
Male, *n* (%)	471 (57)
Caucasian, *n* (%)	815 (98)
Body mass index, kg/m^2^, median (IQR)	29 (25–33)
Chronic heart failure, *n* (%)	501 (60)
Ischemic etiology, *n* (%)	271 (33)
I-II NYHA class 24 h before admission	634 (74)
LVEF, %, mean (SD)	46 (18)
*HFrEF, n (%)*	*263 (32)*
*HFmEF, n (%)*	*207 (25)*
*HFpEF, n (%)*	*360 (43)*
Previous HF admissions within 1 year, mean (SD)	0.9 (1.5)
Newly diagnosed HF, *n* (%)	329 (40)
Hypertension, *n* (%)	635 (77)
Diabetes mellitus, *n* (%)	387 (47)
Atrial fibrillation, *n* (%)	354 (43)
Chronic kidney disease (eGFR < 60 ml/min/1.73 m^2^), *n* (%)	249 (30)
Stroke, *n* (%)	83 (10)
COPD, *n* (%)	131 (16)
Charlson comorbidity index, mean (SD)	3.5 (2.7)
**Clinical data at admission**	
Clinical profile of acute HF, *n* (%)	
*Acutely decompensated chronic HF*	*595 (72)*
*Pulmonary edema*	*115 (14)*
*Right HF*	*34 (4)*
*Others*	*86 (10)*
Heart rate, bpm, median (IQR)	85 (72–100)
Systolic blood pressure, mmHg, median (IQR)	130 (114–150)
**Intravenous therapies**, *n* (%)	
*Diuretics*	*803 (97)*
*Vasodilators, n (%)*	*117 (14)*
*Inotropes*	*47 (6)*
**Clinical data at discharge**	
Heart rate, bpm, median (IQR)	70 (62–80)
Systolic blood pressure, mmHg, median (IQR)	117 (105–130)
Decrease >3 kg of body weight	199 (24)
Length of stay, days, median (IQR)	9 (6–13)
ACEI/ARB, *n* (%)	572 (69)
Beta-blockers, *n* (%)	586 (71)
MRA, *n* (%)	370 (46)
**Outcomes**	
12-month mortality, n (%)	154 (18.6)

*IQR, interquartile range; kg, kilogram; m, meter; NYHA, New York Heart Association; LVEF, left ventricular ejection fraction; SD, standard deviation; HFrEF, heart failure with reduced ejection fraction; HFmEF, heart failure with mid ejection fraction; HFpEF, heart failure with preserved ejection fraction; HF, heart failure; eGFR, estimated glomerular filtration rate; ml, milliliter; min, minute; COPD, chronic obstructive pulmonary disease; bpm, beats per minute; mm, millimeter; ACEI, angiotensin converter enzyme inhibitor; ARB, angiotensin receptor blocker; MRA, mineraloid receptor antagonist.*

### Biomarker Changes During Compensation of Acute Heart Failure Episode

As summarized in Table 2, the biomarkers linked to myocyte stress (NT-proBNP), inflammation (hs-CRP), and matrix remodeling (GDF-15) decreased significantly after the hospital stay whereas the percentage of change of GAL-3 and hs-TnT was negligible. The increase of biomarker reflecting renal damage (Cys-C) was lower than the expected by the biological variability. The Supplementary Table 1 summarizes the best cutoff points of each biomarker predicting the primary outcome according to the ROC analysis.

**TABLE 2:** Time course of biomarkers during the clinical compensation of acute HF.

	**Admission**	**Discharge**	*P*-value*	Delta**
NT-proBNP, ng/L	3710 (1784/7634)	1814 (874/4220)	<0.001	−43.6 (−67.1/−6.7)
Hs-TnT, ng/L	35.2 (20.0/61.9)	34.1 (20.0/60.4)	0.348	−0.9 (−23.5/24.7)
Cystatin C, mg/L	1.5 (1.2/2.0)	1.6 (1.2/2.1)	<0.001	4.1 (−5.5/17.1)
Hs-CRP, mg/L	10.2 (4.5/29.5)	7.4 (3.1/18.8)	<0.001	−34.7 (−66.7/19.5)
GDF-15, ng/L	3366 (2176/5643)	2882 (1963/4989)	<0.001	−11.2 (−30.1/13.0)
GAL-3, mg/L	22.7 (17.1/30.8)	22.1 (16.4/30.8)	0.043	−2.0 (−14.4/14.0)

*Median (1st Quartile/3rd Quartile).*

*Wilcoxon signed rank test with continuity correction.

**Delta: [(discharge value-admission value)/admission value]*100.

*HF, heart failure; NT-proBNP, N-terminal pro-B-type natriuretic peptide; ng, nanograms; L, liter; hsTnT, high sensitivity troponin T; mg, milligrams; hs-CRP, high-sensitivity C-reactive protein; GDF15, Growth/differentiation factor 15; ml, milliliter; GAL-3, galectin-3.*

### Added Prognostic Value of a Multi-Biomarker 2-Point Based Strategy

On top of the best clinical model, the addition of elevated NT-proBNP, hs-CRP, and GDF-15 at admission (>6,319 ng/L, > 15.8 mg/L, and > 5,452 ng/L, respectively), at discharge (>3,239 ng/L, > 12.5 mg/L, and > 4,291 ng/L, respectively), and the inclusion of the magnitude of change during the compensation (–23.3, –21.7, and –15.6%, respectively) gave rise to the highest improvement in the C-index for 12-month mortality (0.782; 95% CI 0.747–0.817, *p* < 0.001). Of notice, the discrimination of this 3-biomarker model was better than that including the six biomarkers, and even better than that based only the biomarkers at discharge. Moreover, the 3-biomarkers model provided a huge reclassification of patients with and without increased risk reaching a statistically significant NRI of 56% for 12-month mortality. These scores were achieved with a correct calibration of the models (Supplementary Figure 1). Similarly, the addition of these 3 biomarkers on top of the MAGGIC, GWTG-HF, Redin-SCORE, and BCN bio-HF models was the best strategy in terms of gain of C-index and reclassification parameters. Table 3 summarizes the discrimination, calibration, IDI, and NRI parameters for the primary outcome given by the clinical models alone, in combination with the 6-biomarker model, and the 3-biomarker strategy. The discrimination capacity of the models for HF-mortality was also better than that for cardiovascular and overall mortality (Supplementary Figure 2).

**TABLE 3:** Added prognostic value of a multi-biomarker 2-point-based risk stratification strategy in acute heart failure to predict 12-month all-cause mortality.

	**C-index**	***P*-value vs. clinical model**	**G-B *p*-value**	**Brier score**	**AIC**	**BIC**	**IDI**	***P*-value for IDI**	**NRI**	***P*-value for NRI**
Clinical model (CM)	0.752 (0.715–0.789)		0.742	0.138	1908	1911				
CM + All biomarker	0.768 (0.730–0.805)	<0.001	0.900	0.134	1887	1899	0.031	<0.001	0.434	<0.001
CM + NT-proBNP, hs-CRP, GDF-15	0.782 (0.747–0.817)	<0.001	0.550	0.133	1889	1919	0.050	<0.001	0.566	<0.001
MAGGIC	0.639 (0.594–0.684)		0.836	0.148	1999	2002				
MAGGIC + All biomarker	0.723 (0.684–0.762)	<0.001	0.995	0.140	1941	1953	0.081	<0.001	0.569	<0.001
MAGGIC + NT-proBNP, hs-CRP, GDF-15	0.745 (0.709–0.782)	<0.001	0.563	0.137	1934	1964	0.108	<0.001	0.661	<0.001
GWTG	0.646 (0.602–0.691)		0.999	0.148	1998	2001				
GWTG + All biomarker	0.722 (0.682–0.762)	<0.001	0.998	0.140	1943	1955	0.078	<0.001	0.575	<0.001
GWTG + NT-proBNP, hs-CRP, GDF-15	0.746 (0.709–0.783)	<0.001	0.053	0.137	1934	1965	0.107	<0.001	0.601	<0.001
Redin-SCORE	0.636 (0.585–0.686)		0.224	0.150	2011	2014				
Redin-SCORE + All biomarker	0.720 (0.680–0.760)	<0.001	0.561	0.140	1944	1956	0.094	<0.001	0.644	<0.001
Redin-SCORE + NT-proBNP, hs-CRP, GDF-15	0.743 (0.706–0.780)	<0.001	0.686	0.137	1936	1967	0.123	<0.001	0.638	<0.001
BCN-BIO HF	0.617 (0.573–0.661)		0.719	0.151	2017	2020				
BCN-BIO HF + All biomarker	0.719 (0.679–0.759)	<0.001	0.882	0.140	1945	1957	0.099	<0.001	0.693	<0.001
BCN-BIO HF + NT-proBNP, hs-CRP, GDF-15	0.743 (0.706–0.780)	<0.001	0.710	0.137	1934	1964	0.132	<0.001	0.694	<0.001

*G-B, Grønnesby and Borgan; AIC, Akaike criteria; BIC, Bayesian criteria; IDI, integrated discrimination improvement; NRI, net reclassification improvement; NT-proBNP, N-terminal pro-B-type natriuretic peptide; hs-CRP, high-sensitivity C-reactive protein; GDF15, growth/differentiation factor 15; CV, cardiovascular; HF, heart failure.*

**FIGURE 1:**
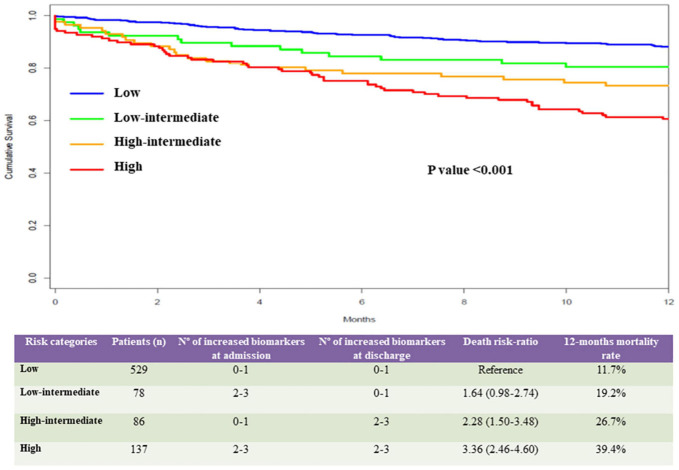
Risk categories based on the values of NT-proBNP, hs-CRP, and GDF-15 in the study population. Upper panel: our study identified 4-risk categories for 12-month all-cause mortality based on the values of NT-proBNP, hs-CRP, and GDF-15: (1) a low-risk category (blue line) included 529 patients presenting none or 1 biomarker above the cutoff values at admission and discharge, (2) a low-intermediate risk category (green line) included 78 patients presenting 2 or 3 elevated biomarkers at admission and none or 1 at discharge, (3) a high-intermediate category (orange line) included 86 patients presenting none or 1 elevated biomarker at admission and 2 or 3 elevated biomarkers at discharge, and (4) a high-risk category (red line) included 137 patients presenting 2 or 3 elevated biomarkers both at admission and discharge. Bottom panel: The 12-month mortality rate for these 4 categories was, respectively, 11.7, 19.2, 26.7, and 39.4%. Considering the low risk category as reference, the mortality risk-ratio was 1.64 (95% CI: 0.98–2.74) for the low-intermediate; 2.28 (95% CI: 1.50–3.48) for the high-intermediate; and 3.36 (95% CI: 2.46–4.60) for the highest risk categories.

The 3-biomarker strategy also allowed to identify 4-risk categories for 12-month all-cause mortality: (1) low-risk group (529 patients) presenting either none or 1 elevated biomarker, (2) low-intermediate risk group (78 patients) presenting 2 or 3 elevated biomarkers at admission but none or 1 at discharge, (3) high-intermediate group (86 patients) presenting either none or 1 elevated biomarker at admission but 2 or 3 at discharge, and (4) high-risk group (137 patients) presenting 2 or 3 elevated biomarkers both at admission and discharge. As shown in the Figure 1, the 12-month mortality rates for these four categories was, respectively, 11.7, 19.2, 26.7, and 39.4% (*p* < 0.001 for the trend). Considering the low risk category as reference, the mortality risk-ratio was 1.64 (95% CI: 0.98–2.74) for the low-intermediate; 2.28 (95% CI: 1.50–3.48) for the high-intermediate; and 3.36 (95% CI: 2.46–4.60) for the highest risk category. Supplementary Table 2 summarizes the predictive capacity gain of all combinations of the six biomarkers, when added to the best clinical model.

## Discussion

### Main Findings

Our study revealed that elevated concentrations of NT-proBNP, hs-CRP, and GDF-15 at hospital admission and discharge in patients with acute HF predicted 12-month mortality better than the best clinical model developed in our population and permitted to define 4 levels of risk. Moreover, the predictive capacity of this 3-biomarker panel was not increased by adding hs-TnT, cystatin C and Galectin-3 in the model and was also superior to the most the currently used scores.

### Predictive Risk Capacity of Biomarker Strategies

Heart failure encompasses several pathophysiological processes that can be indirectly estimated by analyzing the biomarkers specifically related to the underlying mechanisms (Braunwald, 2008). Thus, measurement of a set of biomarkers would afford an integrative knowledge of the complex pathophysiology of HF and, ultimately, would permit a better risk assessment and identification of patients requiring a close follow-up plan. During the last 15 years, at least 20 clinical studies including 3 or more biomarkers have been published (Ishii et al., 2002; van Kimmenade et al., 2006; Januzzi et al., 2007; Rehman et al., 2008; Manzano-Fernández et al., 2009, 2011; Niizeki et al., 2009; Zairis et al., 2010; Pascual-Figal et al., 2011; Shah et al., 2012; Bjurman et al., 2013; Lassus et al., 2013; Lok et al., 2013; Richter et al., 2013; Srinivas et al., 2014; Demissei et al., 2016, 2017a,b; Herrero-Puente et al., 2017; Tromp et al., 2017). As summarized in Supplementary Table 3, half of these reports corresponded to clinical trials (van Kimmenade et al., 2006; Januzzi et al., 2007; Rehman et al., 2008; Manzano-Fernández et al., 2011; Shah et al., 2012; Lok et al., 2013; Demissei et al., 2016, 2017a,b; Tromp et al., 2017), that recruited selected groups of patients, and any case the sample size was greater than that of our study (Ishii et al., 2002; Manzano-Fernández et al., 2009; Niizeki et al., 2009; Zairis et al., 2010; Pascual-Figal et al., 2011; Bjurman et al., 2013; Lassus et al., 2013; Richter et al., 2013; Srinivas et al., 2014; Herrero-Puente et al., 2017). Of notice, in 17 of 20 studies the biomarker was measured either at hospital admission or discharge, and only in 3 cases the biomarkers were measured at both clinical circumstances (Demissei et al., 2016, 2017a,b). The external validity of the data reported in these studies might be hampered by limitations inherent to the *post hoc* analysis in clinical trials, and also to the single-center design in 6 studies (Ishii et al., 2002; Manzano-Fernández et al., 2009; Niizeki et al., 2009; Pascual-Figal et al., 2011; Bjurman et al., 2013; Srinivas et al., 2014).

Our study overcomes some of these limitations and emerges as the first observational, multicenter registry analyzing the capacity of a set of biomarkers double measured at hospital admission and discharge to predict relevant 1-year outcomes in a large group of patients with acute HF. We selected six biomarkers linked to the main processes involved in HF such as neurohormonal activation, myocyte stretch, injury or inflammation, myocardial remodeling and fibrosis, and impaired renal function involved in HF development. Among these 6 biomarkers, we found that the model including the NT-proBNP, hs-CRP, and GDF-15 was the best to predict 12-month mortality. Interestingly, these 3 biomarkers presented the largest magnitude of change from hospital admission to discharge suggesting that patients in whom the linked underlying mechanisms namely myocyte stretch, inflammation, and myocardial remodeling had not improved upon clinical compensation of the HF episode are at high risk of mortality. The percentage of change of the other 3 studied biomarkers hs-cTnT, GAL-3, and Cys-C at discharge was less than 5% and these biomarkers did not improve the discriminative risk capacity beyond that achieved by NT-proBNP, hs-CRP, and GDF-15. The lack of risk prediction of hs-cTnT in our study could deal with several causes. Elevated hs-TnT values in acute HF could be associated to ischemia, inflammation, oxidative stress or impaired renal function. However, all these alterations could also increase the 3 biomarkers, particularly GDF-15 and hs-CRP, already included in the model and, the prognostic role of hs-cTnT could be already covered (Kociol et al., 2010). In addition, we excluded in our study ST-segment elevation acute coronary syndrome as a cause of HF hospitalization and hs-TnT is known to be a strong risk predictor in these patients.

### Clinical Implications

A decrease in the plasma level of natriuretic peptides during the clinical compensation of a HF episode is associated with lower cardiovascular mortality and lower readmissions at 6 months (Savarese et al., 2014). However, a systematic recommendation on their use in clinical practice is not reflected in the current guidelines (Yancy et al., 2013; Ponikowski et al., 2016). Recently, a consensus document of the American Heart Association stated that the measurement of natriuretic peptides, cardiac troponin, and biomarkers of fibrosis at the time of presentation *is useful and reasonable* for establishing prognosis in patients with acutely decompensated HF (Chow et al., 2017). Our study contributes on this important issue by identifying the best combination of 3 out of 6 currently used biomarkers that are the most useful to predict 1-year mortality of patients after hospitalization for heart failure. Specially, the relevant IDI and NRI values by our 3-biomarker model reinforce its role improving the ever-complex HF stratification process.

### Study Limitations

This study includes 98% of Caucasian patients, thus our data might not be fully applicable to other ethnicities or countries. Considering that our study design necessarily required biomarker measurements available at hospital admission and discharge, we did not include patients lacking the discharge sample. Moreover, the size of the study sample did not allow analyzing the performance of the multi-biomarker strategies in subgroups of clinical interest. Therefore, external validation of the clinical model and the full model including biomarkers should be performed.

## Conclusion

In a multicenter, prospective registry of patients with acute HF, we identified 3 out of 6 currently available biomarkers that afforded the highest discriminative power to predict 12-month mortality beyond the best clinical model and also above the currently used MAGGIC, GWTG-HF, Redin-SCORE, and BCN bio-HF scores. Moreover, this simple 3-biomarker panel permitted to define 4 predictive risk levels for 1-year mortality.

What is Known About the Topic?

• The prognostic stratification of patients with acute HF is essential to establish an appropriate personalized follow-up plan.
• Cardiac biomarkers have improved the predictive models of HF patients beyond the already well-established clinical risk predictors.
• Most multi-panel strategies in acute HF have only measured biomarkers in a 1-point time.

What Does This Study Add?

• We evaluate the prognostic role of 6 biomarkers at admission and discharge after HF admission.
• Our study identifies a simple set of 3 biomarkers to predict prognosis of HF patients.
• This panel permits to define 4 predictive risk levels for 12-month mortality.

## Data Availability Statement

The raw data supporting the conclusions of this article will be made available by the authors, without undue reservation.

## Ethics Statement

The protocol was approved by the Ethics Committees of each participating center. The patients/participants provided their written informed consent to participate in this study.

## Author Contributions

JÁ-G, JO-L, and JC contributed to conception and design of the study. JÁ-G and AF-G organized the database. AF-G performed the statistical analysis. JÁ-G wrote the first draft of the manuscript. JÁ-G, ÁG-O, MV-B, AF-G, JO-L, and JC wrote sections of the manuscript. All authors contributed to manuscript revision, read, and approved the submitted version.

## Conflict of Interest

The authors declare that the research was conducted in the absence of any commercial or financial relationships that could be construed as a potential conflict of interest.

## Publisher’s Note

All claims expressed in this article are solely those of the authors and do not necessarily represent those of their affiliated organizations, or those of the publisher, the editors and the reviewers. Any product that may be evaluated in this article, or claim that may be made by its manufacturer, is not guaranteed or endorsed by the publisher.

## Supplementary Material

Supplementary Figure 1 Calibration plots of the clinical, 6-biomarker, and 3-biomarker models for each outcome.

Supplementary Figure 2 Comparison of C-index between clinical, 6-biomarker, and 3-biomarker models for each outcome. Discrimination of the 3-biomarker model (red line) was better than that including only clinical variables (blue line), and even better than that considering all the biomarkers (green line). In addition, C-index for HF-mortality was also better than that for cardiovascular and overall mortality.

## Abbreviations

HF: heart failure
NT-proBNP: N-terminal pro-B-type natriuretic peptide
hs-TnT: high-sensitive T troponin
Cys-C: cystatin-C
GDF-15: growth differentiation factor 15
GAL-3: galectin-3
hs-CRP: high sensitive C reactive protein.

## References

[B1] Álvarez-García J. Ferrero-Gregori A. Puig T. Vázquez R. Delgado J. Pascual-Figal D. (2015). A simple validated method for predicting the risk of hospitalization for worsening of heart failure in ambulatory patients: the Redin-SCORE. *Eur. J. Heart Fail*. 17 818–827. 10.1002/ejhf.287 26011392 PMC5032982

[B2] Bjurman C. Jensen J. Petzold M. Hammarsten O. Fu M. L. X. (2013). Assessment of a multimarker strategy for prediction of mortality in older heart failure patients: a cohort study. *BMJ Open* 3:e002254. 10.1136/bmjopen-2012-002254 23474790 PMC3612770

[B3] Braunwald E. (2008). Biomarkers in heart failure. *N. Engl. J. Med*. 358 2148–2159. 10.1056/NEJMra0800239 18480207

[B4] Chow S. L. Maisel A. S. Anand I. Bozkurt B. de Boer R. A. Felker G. M. (2017). Role of biomarkers for the prevention, assessment, and management of heart failure: a scientific statement from the american heart association. *Circulation* 135 1054–1091. 10.1161/CIR.0000000000000490 28446515

[B5] Demissei B. G. Cotter G. Prescott M. F. Felker G. M. Filippatos G. Greenberg B. H. (2017a). A multimarker multi-time point-based risk stratification strategy in acute heart failure: results from the RELAX-AHF trial. *Eur. J. Heart Fail*. 19 1001–1010. 10.1002/ejhf.749 28133908

[B6] Demissei B. G. Postmus D. Cleland J. G. O’Connor C. M. Metra M. Ponikowski P. (2017b). Plasma biomarkers to predict or rule out early post-discharge events after hospitalization for acute heart failure. *Eur. J. Heart Fail*. 19 728–738. 10.1002/ejhf.766 28251755

[B7] Demissei B. G. Valente M. A. E. Cleland J. G. O’Connor C. M. Metra M. Ponikowski P. (2016). Optimizing clinical use of biomarkers in high-risk acute heart failure patients. *Eur. J. Heart Fail*. 18 269–280. 10.1002/ejhf.443 26634889

[B8] Herrero-Puente P. Prieto-García B. García-García M. Jacob J. Martín-Sánchez F. J. Pascual-Figal D. (2017). Predictive capacity of a multimarker strategy to determine short-term mortality in patients attending a hospital emergency Department for acute heart failure. BIO-EAHFE study. *Clin. Chim. Acta* 466 22–30. 10.1016/j.cca.2017.01.003 28069402

[B9] Ishii J. Nomura M. Nakamura Y. Naruse H. Mori Y. Ishikawa T. (2002). Risk stratification using a combination of cardiac troponin T and brain natriuretic peptide in patients hospitalized for worsening chronic heart failure. *Am. J. Cardiol*. 89 691–695. 11897211 10.1016/s0002-9149(01)02341-4

[B10] Januzzi J. L. Peacock W. F. Maisel A. S. Chae C. U. Jesse R. L. Baggish A. L. (2007). Measurement of the interleukin family member ST2 in patients with acute dyspnea: results from the PRIDE (Pro-brain natriuretic peptide investigation of dyspnea in the emergency department) study. *J. Am. Coll. Cardiol*. 50 607–613. 10.1016/j.jacc.2007.05.014 17692745

[B11] Kociol R. D. Pang P. S. Gheorghiade M. Fonarow G. C. O’Connor C. M. Felker G. M. (2010). Troponin elevation in heart failure prevalence, mechanisms, and clinical implications. *J. Am. Coll. Cardiol*. 56 1071–1078. 10.1016/j.jacc.2010.06.016 20863950

[B12] Lassus J. Gayat E. Mueller C. Peacock W. F. Spinar J. Harjola V. P. (2013). Incremental value of biomarkers to clinical variables for mortality prediction in acutely decompensated heart failure: the multinational observational cohort on acute heart failure (MOCA) study. *Int. J. Cardiol*. 168 2186–2194. 10.1016/j.ijcard.2013.01.228 23538053

[B13] Levy W. C. Mozaffarian D. Linker D. T. Sutradhar S. C. Anker S. D. Cropp A. B. (2006). The seattle heart failure model: prediction of survival in heart failure. *Circulation* 113 1424–1433. 10.1161/CIRCULATIONAHA.105.584102 16534009

[B14] Lok D. J. Klip I. T. Lok S. I. Bruggink-André de la Porte P. W. Badings E. van Wijngaarden J. (2013). Incremental prognostic power of novel biomarkers (growth-differentiation factor-15, high-sensitivity C-reactive protein, galectin-3, and high-sensitivity troponin-T) in patients with advanced chronic heart failure. *Am. J. Cardiol*. 112 831–837. 10.1016/j.amjcard.2013.05.013 23820571

[B15] Lupón J. de Antonio M. Vila J. Peñafiel J. Galán A. Zamora E. (2014). Development of a novel heart failure risk tool: the barcelona bio-heart failure risk calculator (BCN bio-HF calculator). *PLoS One* 9:e85466. 10.1371/journal.pone.0085466 24454874 PMC3893213

[B16] Manzano-Fernández S. Boronat-Garcia M. Albaladejo-Otón M. D. Pastor P. Garrido I. P. Pastor-Pérez F. J. (2009). Complementary prognostic value of cystatin C, N-terminal pro-B-type natriuretic Peptide and cardiac troponin T in patients with acute heart failure. *Am. J. Cardiol*. 103 1753–1759. 10.1016/j.amjcard.2009.02.029 19539088

[B17] Manzano-Fernández S. Mueller T. Pascual-Figal D. Truong Q. A. Januzzi J. L. (2011). Usefulness of soluble concentrations of interleukin family member ST2 as predictor of mortality in patients with acutely decompensated heart failure relative to left ventricular ejection fraction. *Am. J. Cardiol*. 107 259–267. 10.1016/j.amjcard.2010.09.011 21211603 PMC3218083

[B18] McMurray J. J. V. Adamopoulos S. Anker S. D. Auricchio A. Böhm M. Dickstein K. (2012). ESC Guidelines for the diagnosis and treatment of acute and chronic heart failure 2012: the task force for the diagnosis and treatment of acute and chronic heart failure 2012 of the european society of cardiology. developed in collaboration with the heart. *Eur. Heart J*. 33 1787–1847. 10.1093/eurheartj/ehs104 22611136

[B19] Niizeki T. Takeishi Y. Kitahara T. Suzuki S. Sasaki T. Ishino M. (2009). Combination of conventional biomarkers for risk stratification in chronic heart failure. *J. Cardiol*. 53 179–187. 10.1016/j.jjcc.2008.10.003 19304120

[B20] Pascual-Figal D. A. Manzano-Fernández S. Boronat M. Casas T. Garrido I. P. Bonaque J. C. (2011). Soluble ST2, high-sensitivity troponin T- and N-terminal pro-B-type natriuretic peptide: complementary role for risk stratification in acutely decompensated heart failure. *Eur. J. Heart Fail*. 13 718–725. 10.1093/eurjhf/hfr047 21551163

[B21] Peterson P. N. Rumsfeld J. S. Liang L. Albert N. M. Hernandez A. F. Peterson E. D. (2010). A validated risk score for in-hospital mortality in patients with heart failure from the american heart association get with the guidelines program. *Circ. Cardiovasc. Qual. Outcomes* 3 25–32. 10.1161/CIRCOUTCOMES.109.854877 20123668

[B22] Pocock S. J. Ariti C. A. McMurray J. J. V. Maggioni A. Køber L. Squire I. B. (2013). Predicting survival in heart failure: a risk score based on 39 372 patients from 30 studies. *Eur. Heart J*. 34 1404–1413. 10.1093/eurheartj/ehs337 23095984

[B23] Ponikowski P. Voors A. A. Anker S. D. Bueno H. Cleland J. G. F. Coats A. J. S. (2016). 2016 ESC Guidelines for the diagnosis and treatment of acute and chronic heart failure: the task force for the diagnosis and treatment of acute and chronic heart failure of the European society of cardiology (ESC)Developed with the special contribution of the heart failure association (HFA) of the ESC. *Eur. Heart J*. 37 2129–2200. 10.1093/eurheartj/ehw128 27206819

[B24] Rehman S. U. Mueller T. Januzzi J. L. (2008). Characteristics of the novel interleukin family biomarker ST2 in patients with acute heart failure. *J. Am. Coll. Cardiol*. 52 1458–1465. 10.1016/j.jacc.2008.07.042 19017513

[B25] Richter B. Koller L. Hohensinner P. J. Zorn G. Brekalo M. Berger R. (2013). A multi-biomarker risk score improves prediction of long-term mortality in patients with advanced heart failure. *Int. J. Cardiol*. 168 1251–1257. 10.1016/j.ijcard.2012.11.052 23218577

[B26] Savarese G. Musella F. D’Amore C. Vassallo E. Losco T. Gambardella F. (2014). Changes of natriuretic peptides predict hospital admissions in patients with chronic heart failure: a meta-analysis. *JACC Heart Fail*. 2 148–158. 10.1016/j.jchf.2013.11.007 24720923

[B27] Shah R. V. Truong Q. A. Gaggin H. K. Pfannkuche J. Hartmann O. Januzzi J. L. (2012). Mid-regional pro-atrial natriuretic peptide and pro-adrenomedullin testing for the diagnostic and prognostic evaluation of patients with acute dyspnoea. *Eur. Heart J*. 33 2197–2205. 10.1093/eurheartj/ehs136 22645194 PMC3432234

[B28] Srinivas P. Manjunath C. N. Banu S. Ravindranath K. S. (2014). Prognostic significance of a multimarker strategy of biomarkers in acute heart failure. *J. Clin. Diagn. Res*. 8 MC01–MC06. 10.7860/JCDR/2014/9289.4783 25386472 PMC4225924

[B29] Tromp J. Khan M. A. F. Klip I. T. Meyer S. de Boer R. A. Jaarsma T. (2017). Biomarker profiles in heart failure patients with preserved and reduced ejection fraction. *J. Am. Heart Assoc*. 6:e003989. 10.1161/JAHA.116.003989 28360225 PMC5532986

[B30] van Kimmenade R. R. Januzzi J. L. Ellinor P. T. Sharma U. C. Bakker J. A. Low A. F. (2006). Utility of amino-terminal pro-brain natriuretic peptide. *J. Am. Coll. Cardiol*. 48 1217–1224. 10.1016/J.JACC.2006.03.061 16979009

[B31] van Vark L. C. Lesman-Leegte I. Baart S. J. Postmus D. Pinto Y. M. Orsel J. G. (2017). Prognostic value of serial ST2 measurements in patients with acute heart failure. *J. Am. Coll. Cardiol*. 70 2378–2388. 10.1016/j.jacc.2017.09.026 29096809

[B32] Yancy C. W. Jessup M. Bozkurt B. Butler J. Casey D. E. Jr. Drazner M. H. (2013). 2013 ACCF/AHA guideline for the management of heart failure. *Circulation* 128 e240–e327. 10.1161/CIR.0b013e31829e8776 23741058

[B33] Zairis M. N. Tsiaousis G. Z. Georgilas A. T. Makrygiannis S. S. Adamopoulou E. N. Handanis S. M. (2010). Multimarker strategy for the prediction of 31 days cardiac death in patients with acutely decompensated chronic heart failure. *Int. J. Cardiol*. 141 284–290. 10.1016/j.ijcard.2008.12.017 19157603

