# Supplementary material for: A 3-Biomarker 2-Point-Based Risk Stratification Strategy in Acute Heart Failure
Source: Front Physiol. 2021 Oct 22;12:708890. doi: 10.3389/fphys.2021.708890 (PMC8569896; doi:10.3389/fphys.2021.708890)
Supplement: Supplementary Figure 1 — Calibration plots of the clinical, 6-biomarker, and 3-biomarker models for each outcome. [file Presentation_2.PPTX]

## Slide 1
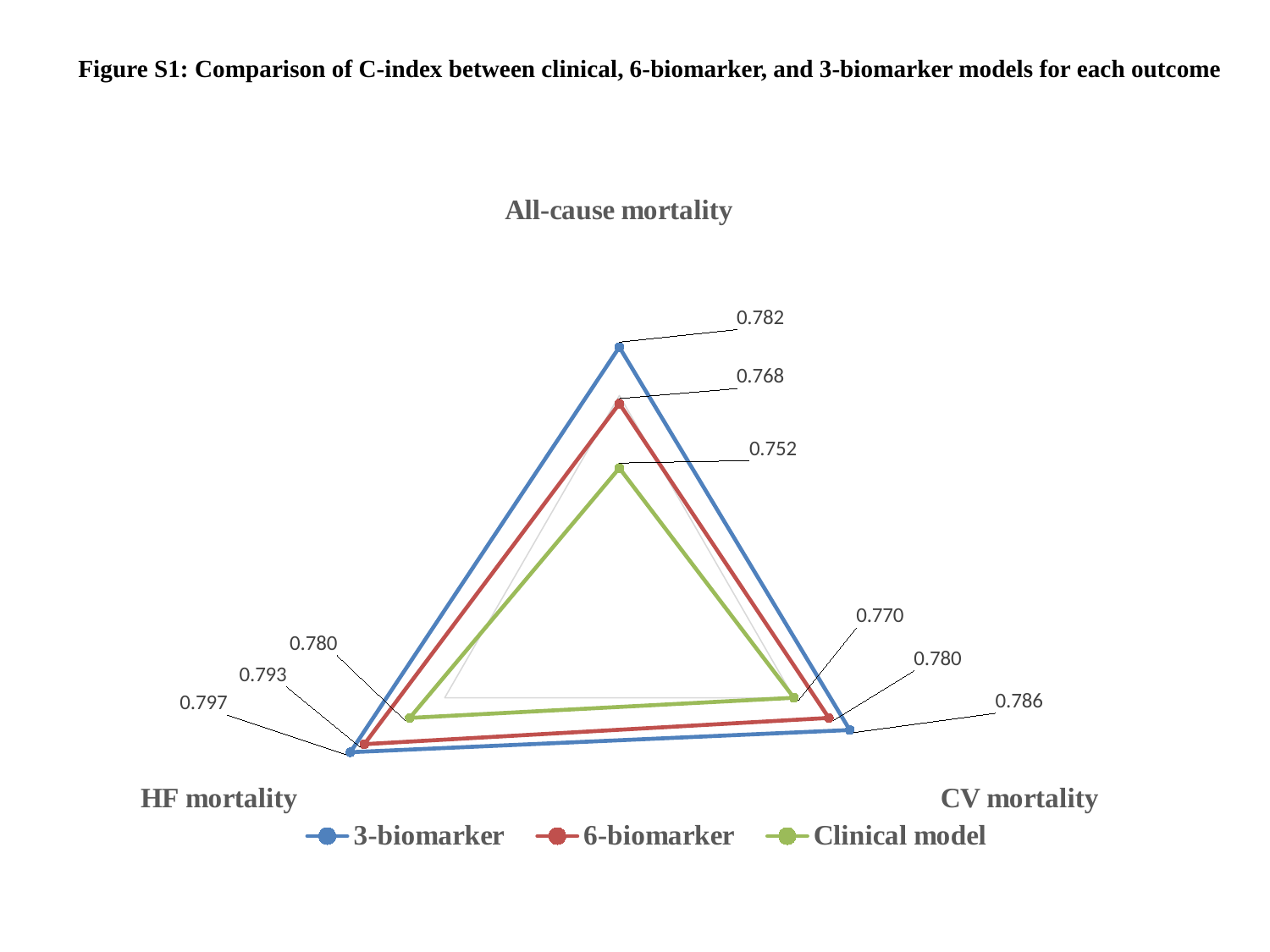

Figure S1: Comparison of C-index between clinical, 6-biomarker, and 3-biomarker models for each outcome
### Chart
| Category | 3-biomarker | 6-biomarker | Clinical model |
|---|---|---|---|
| All-cause mortality | 0.782 | 0.768 | 0.752 |
| CV mortality | 0.786 | 0.78 | 0.77 |
| HF mortality | 0.797 | 0.793 | 0.78 |
