# Supplementary material for: A 3-Biomarker 2-Point-Based Risk Stratification Strategy in Acute Heart Failure
Source: Front Physiol. 2021 Oct 22;12:708890. doi: 10.3389/fphys.2021.708890 (PMC8569896; doi:10.3389/fphys.2021.708890)
Supplement: Supplementary Figure 2 — Comparison of C-index between clinical, 6-biomarker, and 3-biomarker models for each outcome. Discrimination of the 3-biomarker model (red line) was better than that including only clinical variables (blue line), and even better than that considering all the biomarkers (green line). In addition, C-index for HF-mortality was also better than that for cardiovascular and overall mortality. [file Presentation_1.PPTX]

## Slide 1
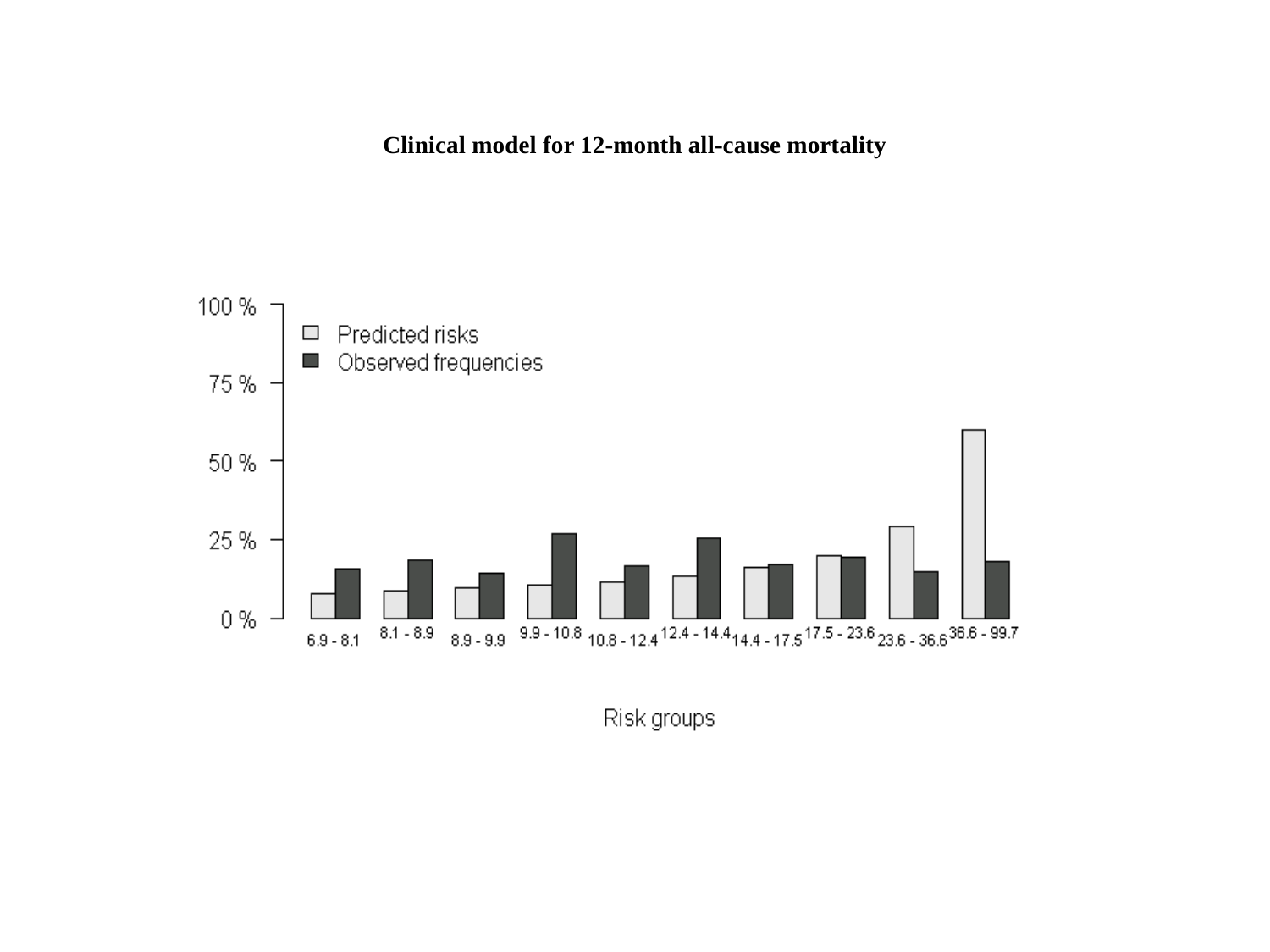

Clinical model for 12-month all-cause mortality

## Slide 2
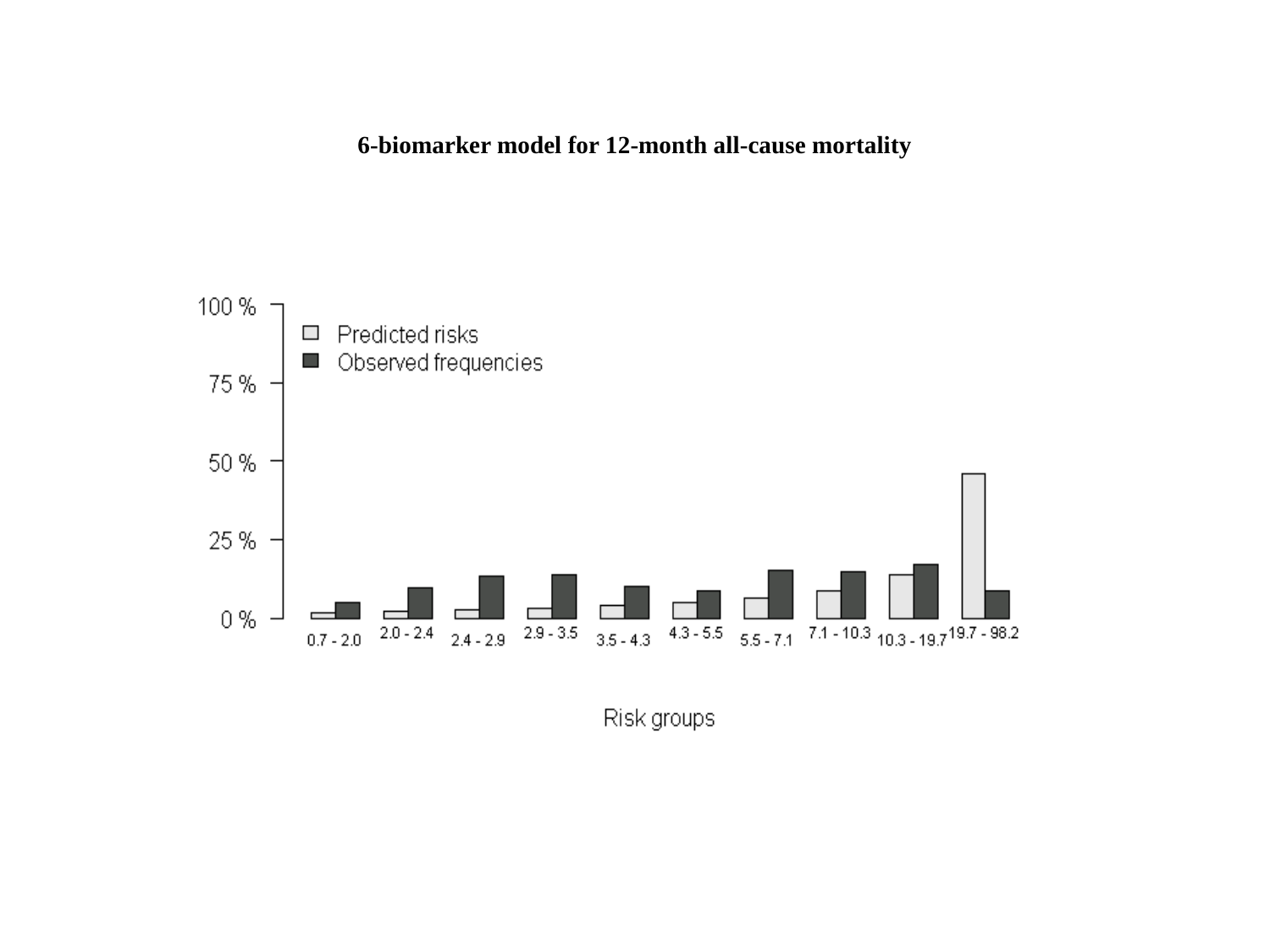

6-biomarker model for 12-month all-cause mortality

## Slide 3
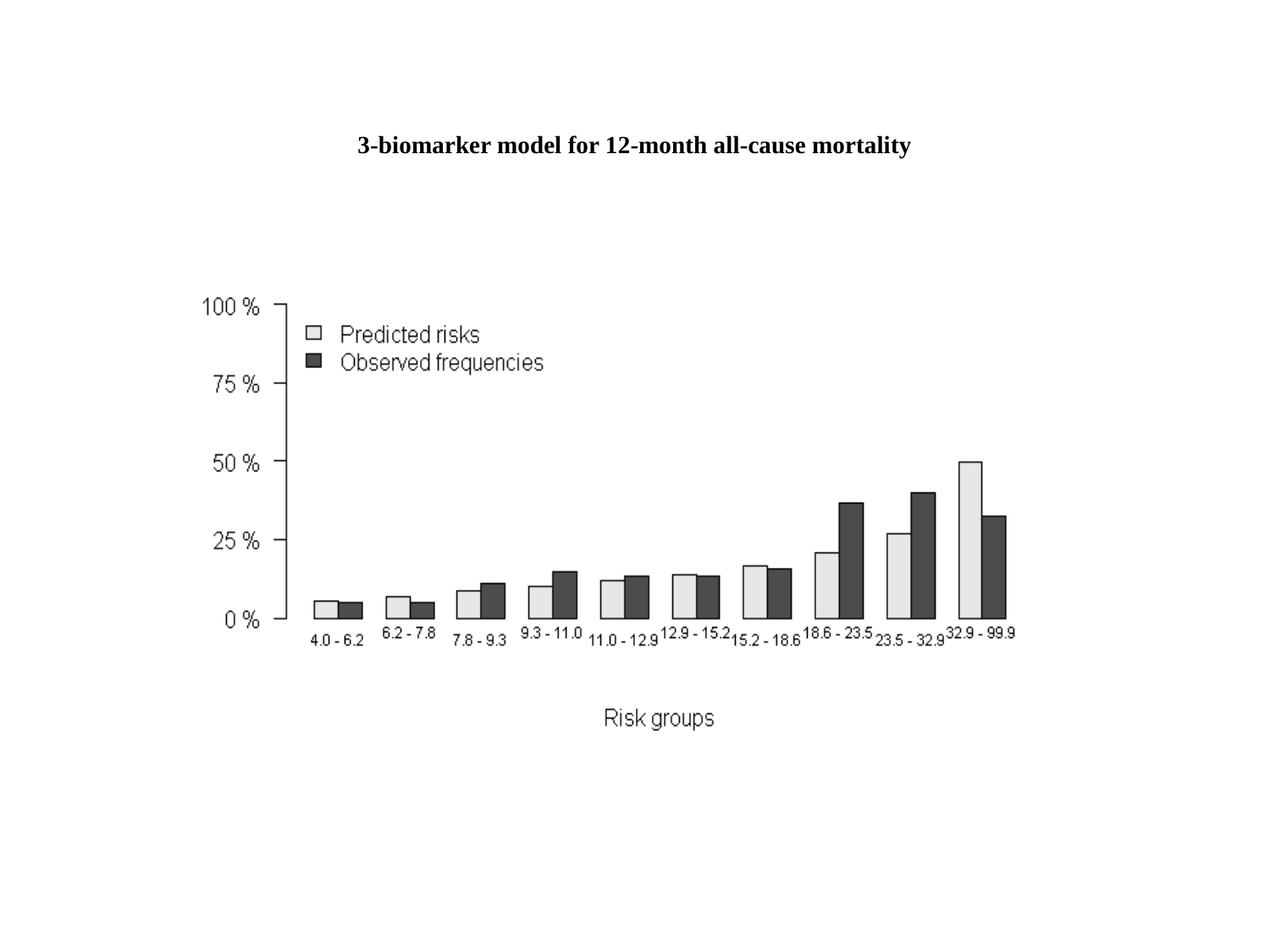

3-biomarker model for 12-month all-cause mortality

## Slide 4
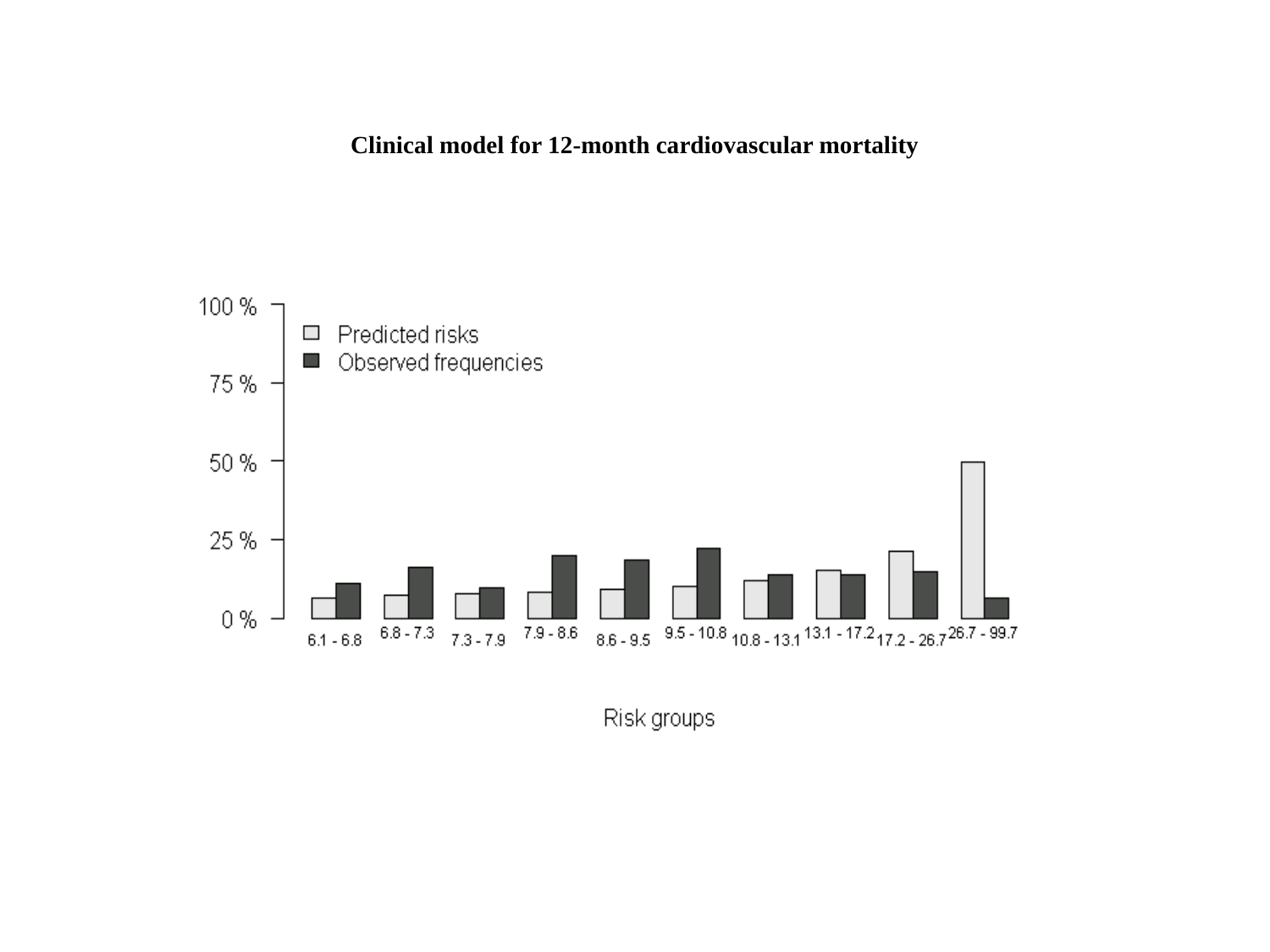

Clinical model for 12-month cardiovascular mortality

## Slide 5
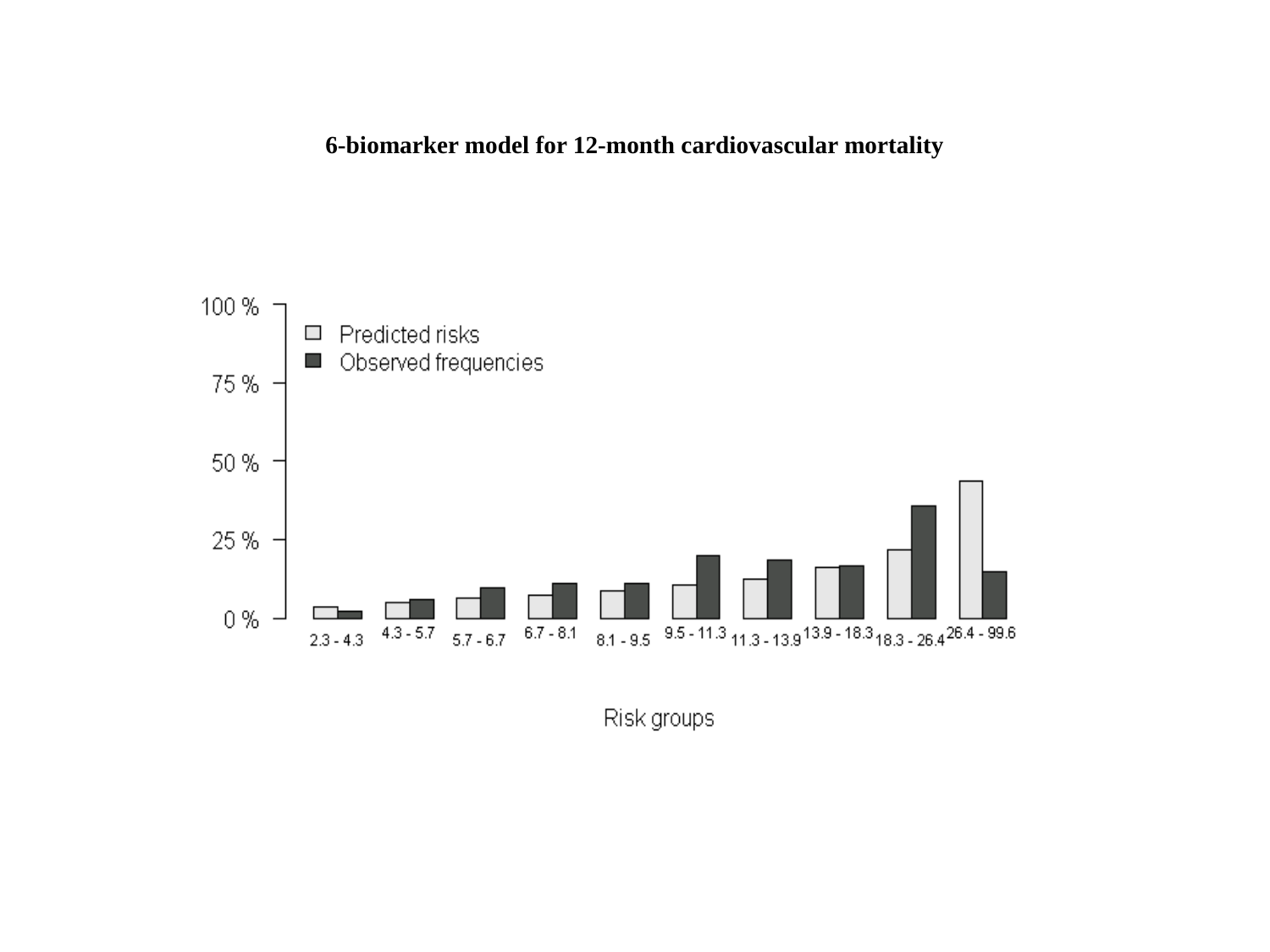

6-biomarker model for 12-month cardiovascular mortality

## Slide 6
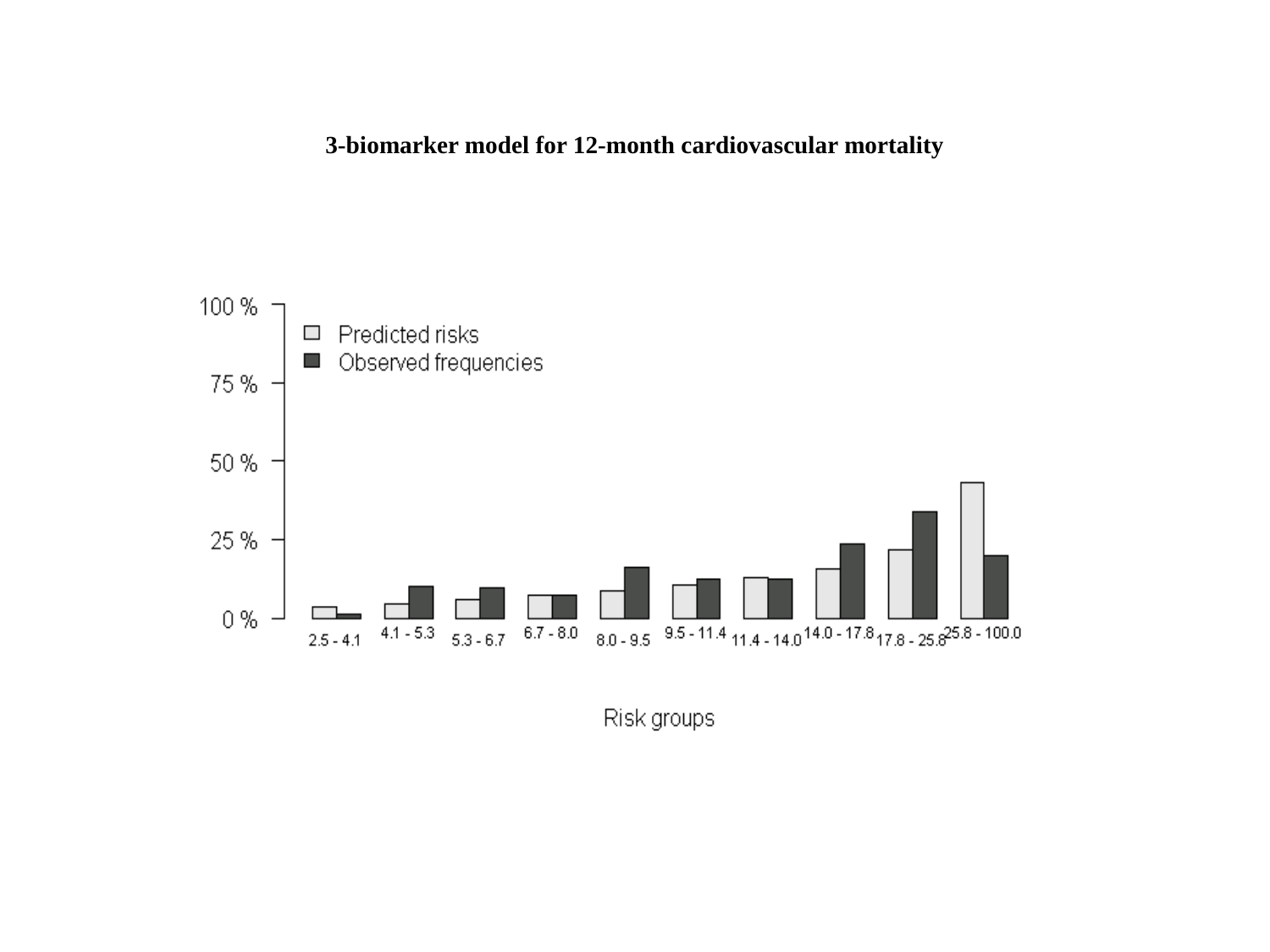

3-biomarker model for 12-month cardiovascular mortality

## Slide 7
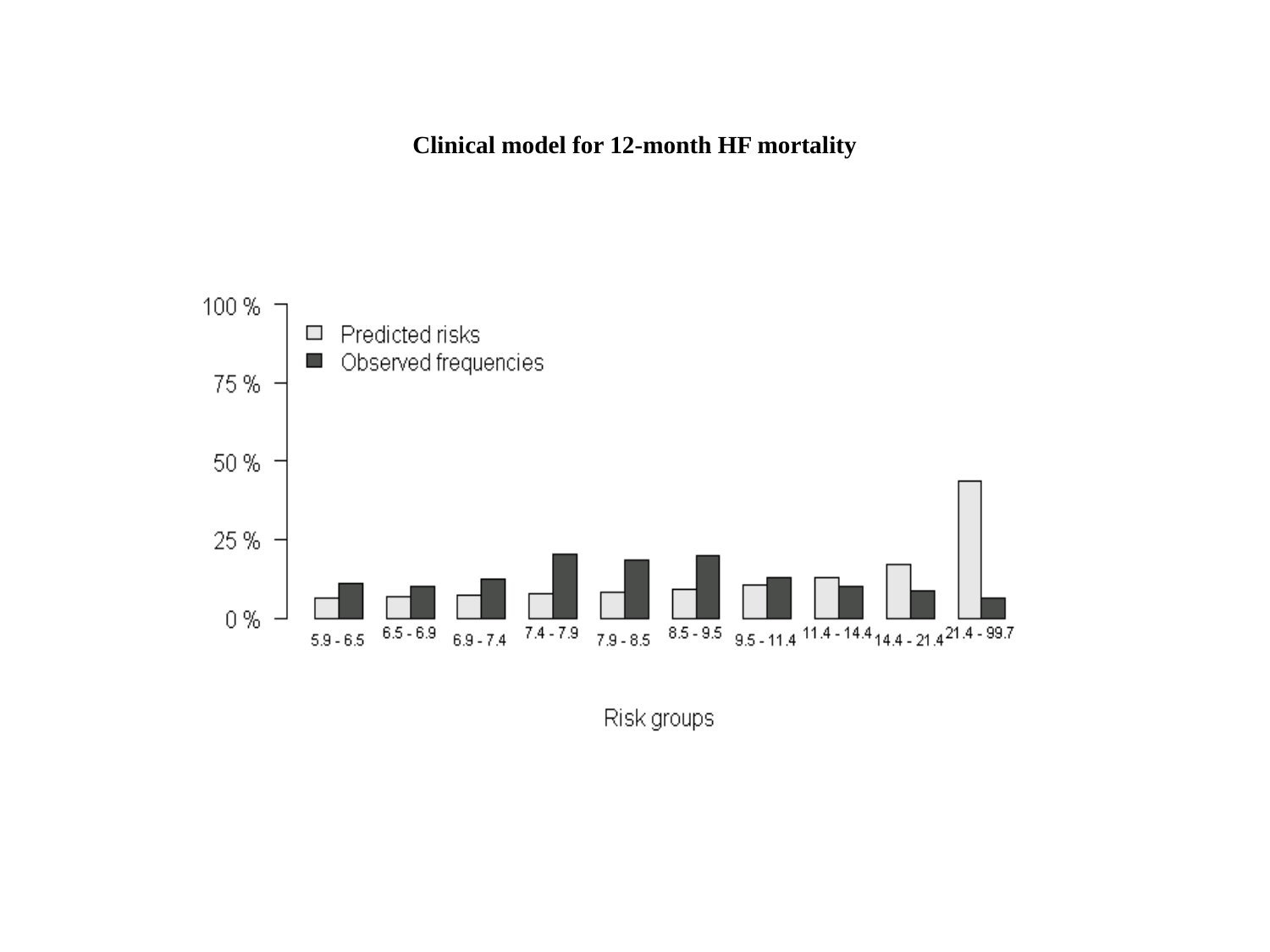

Clinical model for 12-month HF mortality

## Slide 8
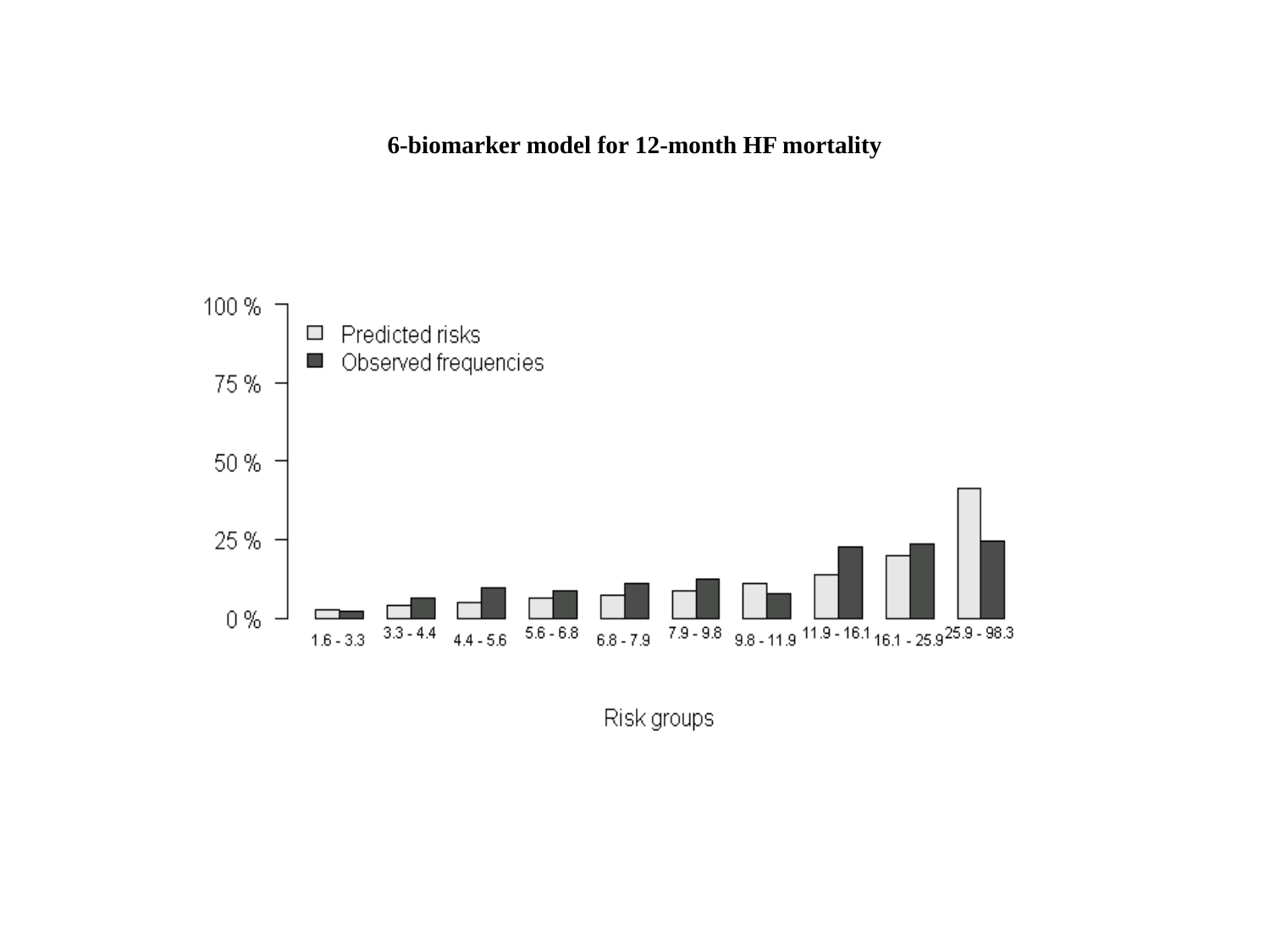

6-biomarker model for 12-month HF mortality

## Slide 9
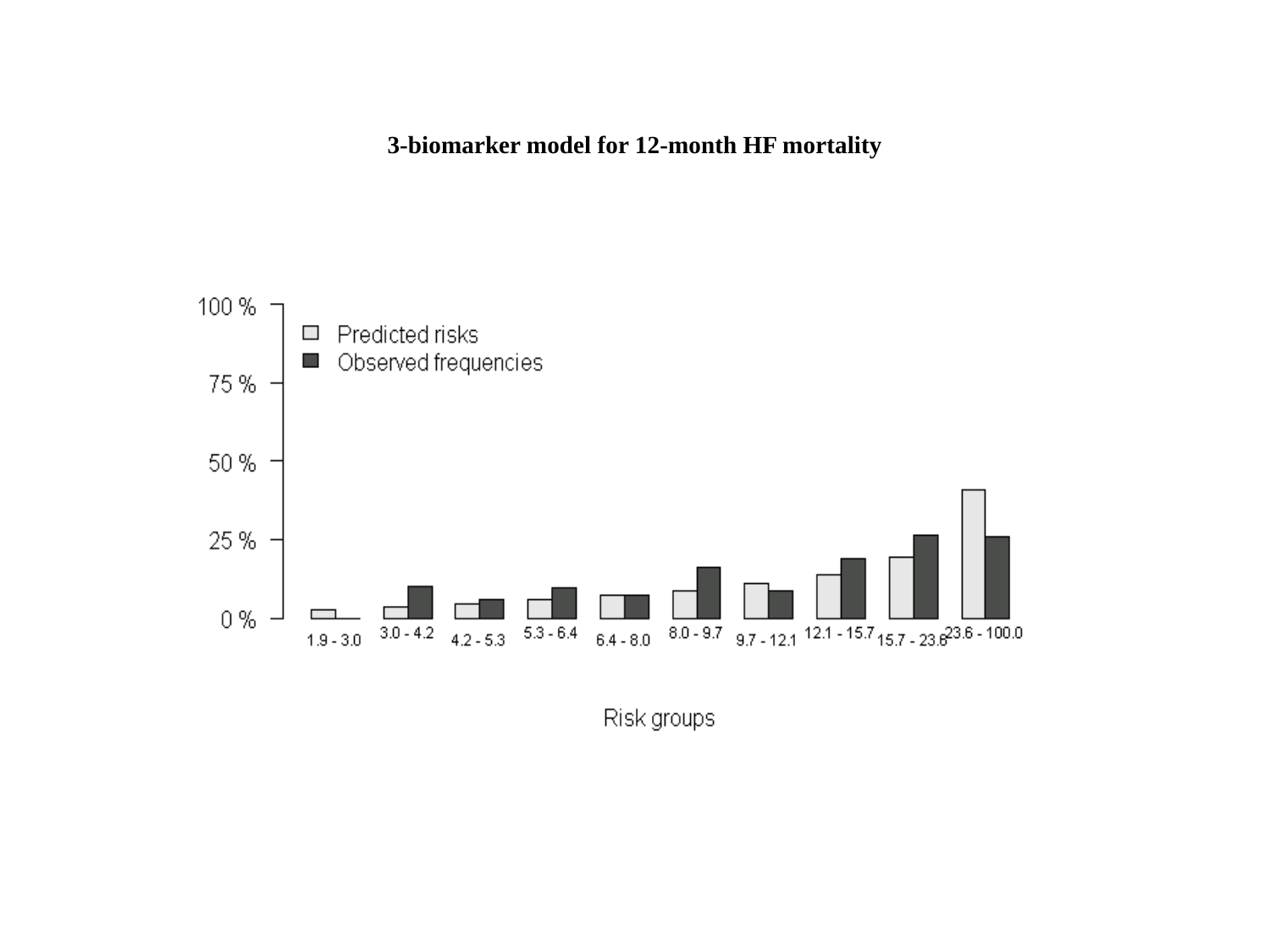

3-biomarker model for 12-month HF mortality

## Slide 10
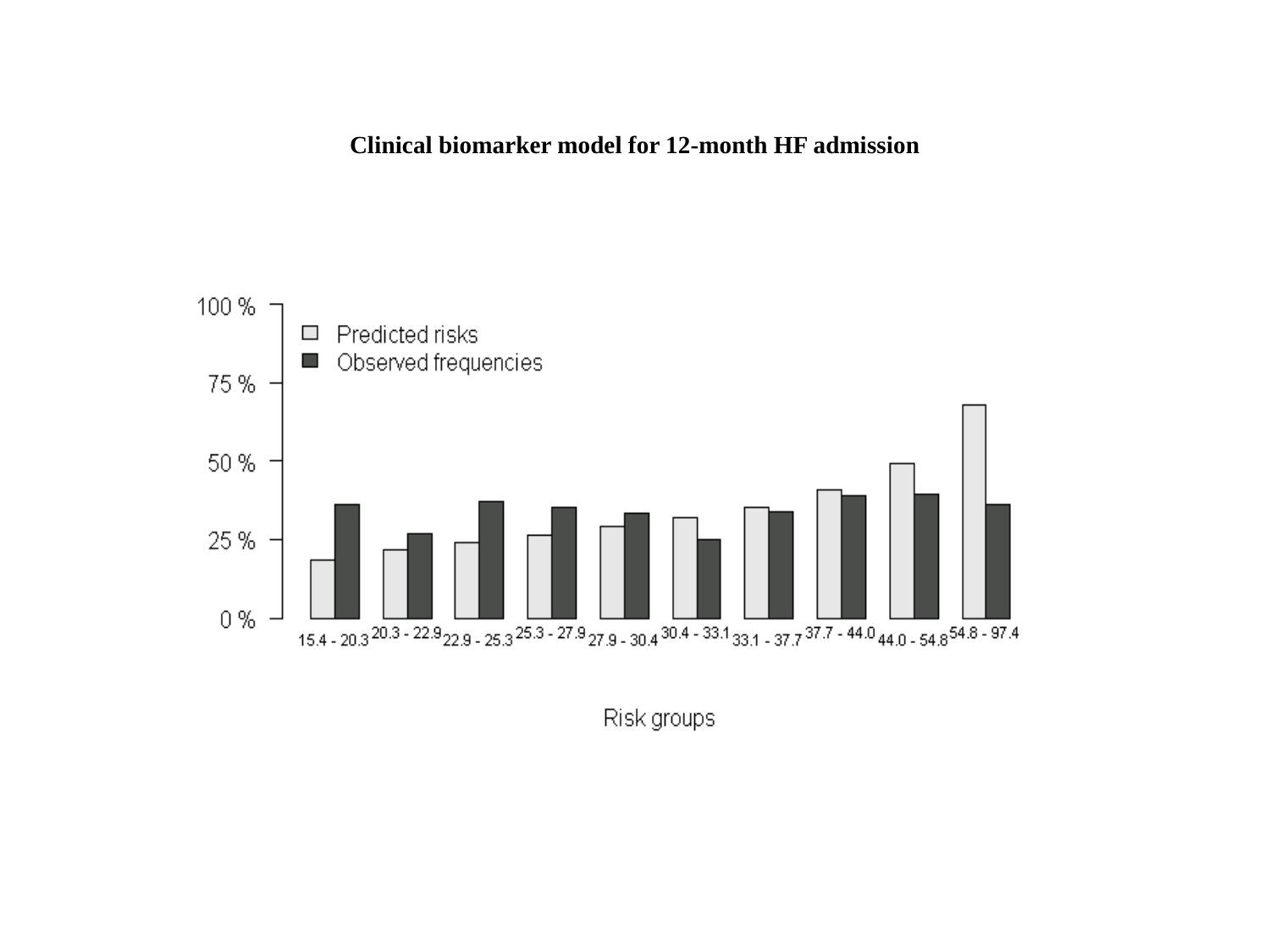

Clinical biomarker model for 12-month HF admission

## Slide 11
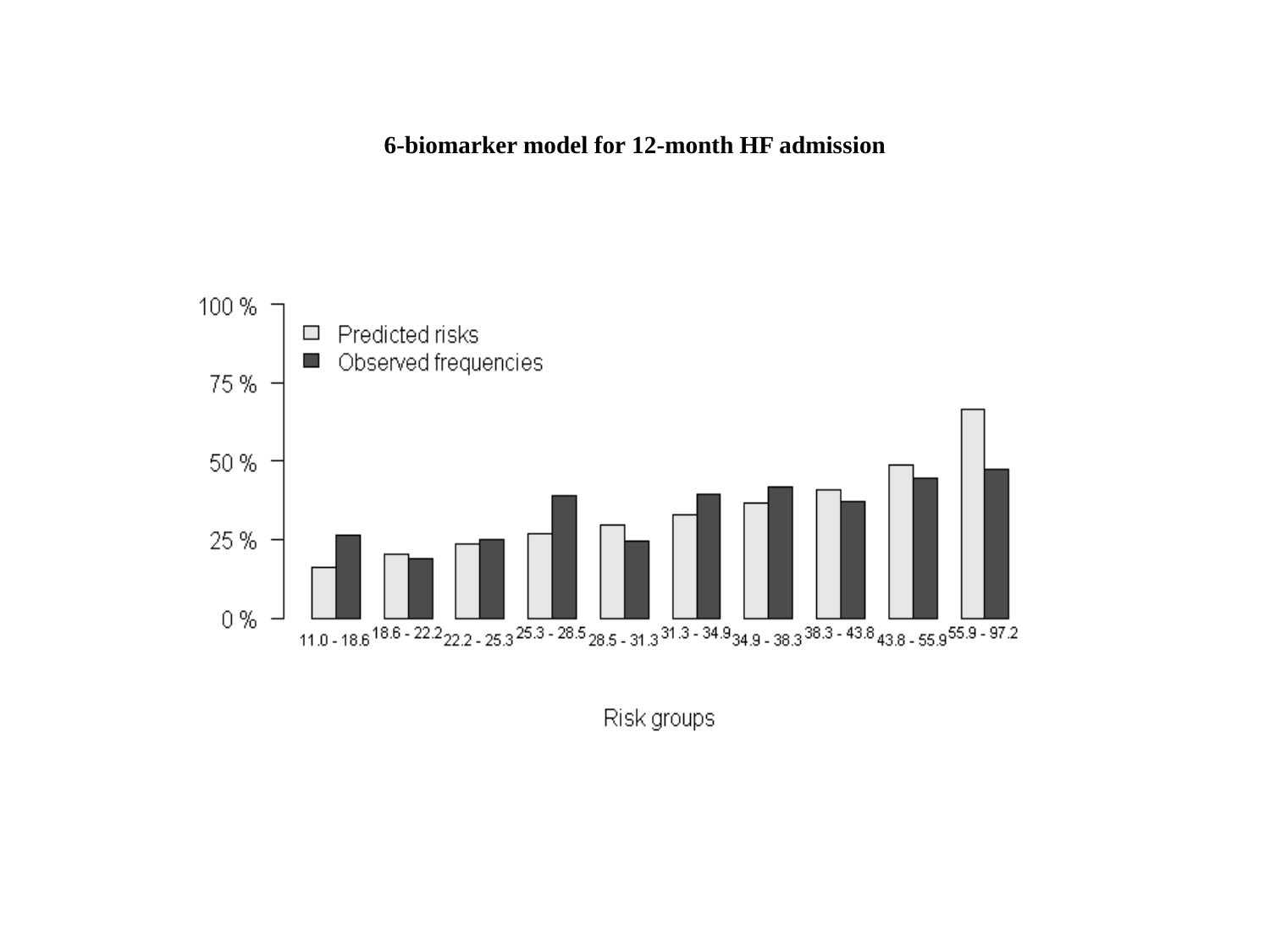

6-biomarker model for 12-month HF admission

## Slide 12
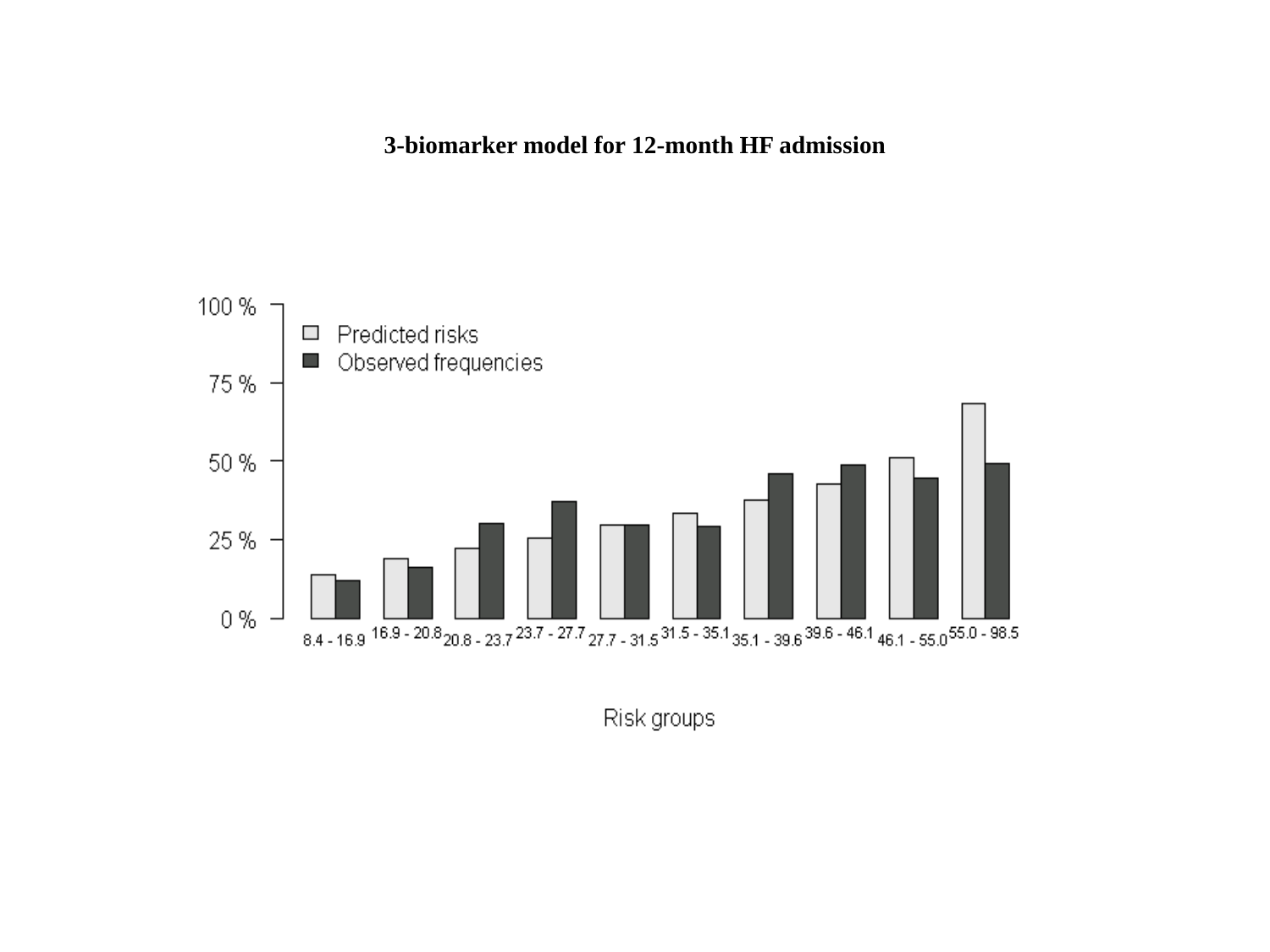

3-biomarker model for 12-month HF admission
