# Supplementary material for: A 3-Biomarker 2-Point-Based Risk Stratification Strategy in Acute Heart Failure
Source: Front Physiol. 2021 Oct 22;12:708890. doi: 10.3389/fphys.2021.708890 (PMC8569896; doi:10.3389/fphys.2021.708890)
Supplement: Supplementary file 3 [file Data_Sheet_1.PDF]

**Supplementary appendix #1**

# LIST OF REDINSCOR II VARIABLES

## 1. Demographic and previous clinical history

Sex

Date of birth

Age

Previous history of heart failure (HF)

Years of evolution since the first diagnosis of HF

Number of previous hospitalizations due to cardiovascular causes (last 12 months)

Number of previous hospitalizations due to HF (last 12 months)

Usual place of previous clinical checks

HF etiology

Smoker status

History of Alcoholism

History of Dyslipidemia

History of Diabetes Mellitus

History of Hypertension

Previous Peripheral Vascular Disease

Anemia

Chronic obstructive pulmonary disease

Stroke

Chronic kidney disease

Charlson index

Barthel index

Pfeiffer index

Previous myocardial infarction

Atrial fibrillation

Previous Syncope

Previous revascularization and type

Previous Pacemaker

Previous Resynchronization therapy

Previous Implantable converter defibrillator

**2. Case history and physical examination**

Cause of current admission

Length of stay

NYHA class functional

Reduced exercise tolerance

Orthopnea

Paroxysmal Nocturnal Dyspnea

Bilateral ankle edema

Third sound gallop

Rales

Neck vein distension

Hepatomegaly

Hepatojugular Reflux

Ascitis

Heart rate

Systolic blood pressure

Diastolic blood pressure

Weight

Height

Body mass index

Waist circumference

**3. Chest radiography**

Cardiothoracic index

Congestion signs on X-ray

**4. ECG data at admission and discharge**

RR interval

Rhythm

PR interval

Left bundle branch block

Right bundle branch block

QRS duration

Left ventricular hypertrophy signs

Q waves

QT interval

Corrected QT interval

**5. Echocardiographic data**

Left ventricular end-diastolic diameter

Left ventricular end-systolic diameter

LVEF

Septum Thickness

Thickness of LV posterior wall

Left atrial diameter

Right ventricular end-diastolic diameter

TAPSE

Mitral regurgitation

Pattern Diastolic filling

**6. Laboratory blood test at admission and discharge**

Hemoglobin

Sodium

Potassium

Proteins

Albumin

Glucose

Urea

Creatinine

Total cholesterol

HDL-cholesterol

LDL-cholesterol

Triglycerides

Aspartate aminotransferase

Alanine aminotransferase

Gamma-glutamyl transpeptidase

TSH

Hs-Troponin T

Pro-BNP

GDF-15

Gal-3

Cys C

Hs-CRP

**7. Previous medical treatment and at discharge**

ACE inhibitors

Aldsterone receptor blockers

Beta-blockers

Dihydropyridine calcium channel blockers

Non-Dihydropyridine calcium channel blockers

Antithrombotic agents and anticoagulants

Statins

Oral Antidiabetics

Loop diuretics

Thiazide diuretics

Spironolactone

Eplerenone

Antiarrhythmics

Nitrates

Hydralazine

Treatment for anemia

Other drugs

**8. Treatment in the first 24 hours of admission**

Stay unit

Dose of furosemide and mode of administration

Ultrafiltration

Inotropic / vasoactive agents / intravenous vasodilators

Type of respiratory support

Type of mechanical support

Bladder catheter

Swan-Ganz catheter

**Supplementary appendix #2**

*** The investigators of the Spanish Heart Failure Network (REDINSCOR II):**

**Hospital General Universitario Gregorio Marañón:** F. Fernández-Avilés, M. Abeytua, P. Ancillo, A. Arenal, F. Atienza, J. Bermejo, J. Blanco, H. Bueno, T. Datino, J. Elízaga, J. Fernández Yáñez, L. Fernández Pena, M.E. Fernández Santos, J.A. García Robles, A. González Mansilla, A. González Pinto, E. González Torrecilla, E. Gutiérrez Ibañes, M. Juárez, J. López Mesa, M.J. Lorenzo, A. Martínez Climent, M. Martínez-Sellés, J. Palomo, E. Pérez David, R. Sanz, F. Sarnago, J. Soriano, I. Sousa, M. E. Vázquez Álvarez, A. Vázquez Castaño, A. Villa, E. Villacorta, R. Yotti.

**Hospital Clínic de Barcelona**: J. Brugada, M. Batlle, A. Berruezo, S. Hevia, L. Mont, F. Pérez-Villa; **Hospital de la Santa Creu i Sant Pau**: J. Cinca, E. Roig, A. Bayés de Luna, X. Borrás, F. Carreras, A. Ferrero-Gregori, J.M. Guerra, L. Hove-Madsen, E. Jorge, J. Padró, T. Puig, X. Viñolas, A. Mendez, J. Alvarez-Garcia, M. Vives-Borras; Eduard Sole-Gonzalez, Mercedes Rivas-Lasarte; Lidia Bos Real; A. Sionis; L. López;

**Hospital Santa Creu i Sant Pau-Bioquímica:** J. Ordóñez-Llanos, S. Benítez, R. Bonet, M. Estruch, M. Martinez Bujidos, J. Mercé, M. Pérez Cuellar, A. Rull, J.L. Sánchez Quesada.

**Hospital Clínico Universitario de Santiago de Compostela**: J.R. González-Juanatey, M. Bandín, S. Eiras, L. Fernández-Hernández, J. García-Acuña, I. Gómez-Otero, L. Grigorian-Shamagian, F. Lago, P. Manzón, M. Moure, F. Otero-Raviña, F. Otero-Santiago, B. K. Rodino Janeiro, J. Rubio, A. Salgado, A. Seoane, A. Varela, P. V. Lear;

**Hospital Clínico San Carlos – Cardiovascular Insitute:** J. Pérez-Villacastín, R. Bover, M. Cobos, J. García-Quintanilla, J. Moreno, N. Pérez-Castellano, M. Pérez-Serrano, I. Vila; **Hospital 12 de Octubre:** J. F. Delgado, F. Arribas, P. Escribano, A.

Flox, C. Jiménez López-Guarch, M. Paradina, J. Ruiz-Cano, C. Sáenz de la Calzada, R. Salguero, V. Sánchez-Sánchez, R. Tello de Meneses, M. Vicente-Hernández; **Hospital Puerta de Hierro:** L. Alonso-Pulpón, I. Fernández -Lozano, P. García-Pavía, A. García-Touchard, M. Gómez-Bueno, J. Márquez, J. Segovia, L. Silva, M. Vázquez-Mosquera; **Hospital Universitario Virgen de la Arrixaca:** M. Valdés, A. García-Alberola, I. Garrido, D. A. Pascual-Figal, F. J. Pastor-Pérez, J. Sánchez-Más, P. Tornel; **Hospital La Fe:** M. Rivera, E. Roselló-Lleti, M. Portolés, E. Tarazón, A. Ortega, M. Molina-Navarro, L. Martínez-Dolz, A. Salvador, L. Almenar; **Hospital Universitario Virgen de Valme:** R. Vázquez, J. Cubero, A. Fernández-Palacín, D. García-Medina, S. García-Rey, E. Laguna, J. Leal del Ojo, F. Miñano, L. Pastor-Torres, R. Pavón, A. Pérez-Navarro, D. Villagómez; **Hospital Universitario Puerta del Mar:** R. Vázquez, R. Arana, D. Bartolomé, P. Cabeza, G. Calle-Pérez, F. Camacho, L. Cano, A. Carrillo, E. Díaz-Retamino, V. Escolar, R. Fernández-Rivero, S. Gamaza, A. Giráldes, N. Hernández-Vicente, M. Lagares, J. López-Benítez, M. Marante, E. Otero, J. Pedregal, M. Sancho-Jaldón, R. Sevillano , R. Zayas; **Hospital Universitario Arnau de Vilanova:** F. Worner, L. Barta, C. Bravo, J. Cabau, J. Casanova, B. Daga, I. De la Puerta, I. Hernández-Martín, E. Piñol, E. Pueo, G. Torres, A. Troncoso, D. Viles; **Hospital Universitario Joan XXIII:** A. Bardají, J. Mercè, E. Sanz-Girgas, P. Valdovinos, I. Serrano; **Hospital Son Espases:** A. Grau-Sepúlveda, C. Fiol, P. Pericas, M. Villalonga. **Hospital Universitario A Coruña:** A. Castro-Beiras†, J.M. Vázquez Rodríguez, E. Barge Caballero, R. Barriales Villa, A. Bouzas, L. Cazón, M.G. Crespo Leiro, X.G. Fernández Fernádez, Z.M. Grillé, M. Hermida, R. Marzoa, L. Monserrat, J. Muñiz, L. Núñez Fernández, M.J. Paniagua, T.R. Pérez Castro, J. Peteiro, T. Ripoll Vera, M.I. Rodríguez García, N. Suárez, N. Vázquez González.

**Hospital Universitario de Salamanca:** P.L. Sánchez Fernández, A. Arribas Jiménez, C. Avila, M. Cascón, I. Cruz, F. del Campo, A. Diego Nieto, M. Diego Dominguez, A. Iscar, J. Jiménez Candíl, F. Martín Herrero, J. Martín Moreiras, A. Martín García, J.L. Moriñigo, P. Pabón, J. Rodríguez Collado.
