# Supplementary material for: A 3-Biomarker 2-Point-Based Risk Stratification Strategy in Acute Heart Failure
Source: Front Physiol. 2021 Oct 22;12:708890. doi: 10.3389/fphys.2021.708890 (PMC8569896; doi:10.3389/fphys.2021.708890)
Supplement: Supplementary file 4 [file Data_Sheet_2.docx]

**Supplementary Table S1: Cutoff points of each biomarker to predict the 12-month mortality based on the ROC analysis**

|  | **Cutoff value** | **Sensitive (%)** | **Specificity (%)** | **AUC (95% CI)** |
| --- | --- | --- | --- | --- |
| **NT-proBNP** | | | | |
| **Admission, ng/L** | 6319 | 49.4 | 72.3 | 61.3 (56.3-66.4) |
| **Discharge, ng/L** | 3239 | 58.4 | 72.6 | 69.0 (64.3-73.7) |
| **Delta (%)** | -23.3 | 55.8 | 70.5 | 64.9 (60.3-69.5) |
| **Hs-TnT** | | | | |
| **Admission, ng/L** | 37.4 | 64.3 | 57.2 | 62.6 (57.9-67.3) |
| **Discharge, ng/L** | 36.0 | 66.9 | 57.7 | 62.8 (58.1-67.6) |
| **Delta (%)** | -32.2 | 20.1 | 83.3 | 49.2 (44.0-54.3) |
| **Cystatin C** | | | | |
| **Admission, mg/L** | 1.8 | 51.9 | 74.1 | 65.5 (60.6-70.3) |
| **Discharge, mg/L** | 1.6 | 75.3 | 53.6 | 66.6 (61.9-71.3) |
| **Delta (%)** | 14.2 | 35.1 | 72.2 | 51.7 (46.4-57.0) |
| **Hs-CRP, mg/L** | | | | |
| **Admission, mg/L** | 15.8 | 42.2 | 63.5 | 51.8 (46.7-56.9) |
| **Discharge, mg/L** | 12.5 | 44.8 | 68.6 | 57.9 (52.9-62.8) |
| **Delta (%)** | -21.7 | 52.6 | 61.2 | 57.9 (52.9-62.8) |
| **GDF-15** | | | | |
| **Admission, ng/L** | 5452 | 51.3 | 78.7 | 66.5 (61.5-71.4) |
| **Discharge, ng/L** | 4291 | 54.5 | 74.6 | 67.8 (63.1-72.6) |
| **Delta (%)** | -15.6 | 66.9 | 46.2 | 53.7 (48.5 – 58.9) |
| **GAL-3** | | | | |
| **Admission, mg/L** | 27.4 | 50.0 | 70.1 | 60.4 (55.2-65.6) |
| **Discharge, mg/L** | 24.7 | 57.1 | 63.0 | 60.3 (55.1-65.4) |
| **Delta (%)** | 3.3 | 64.3 | 42.2 | 52.3 (47.2-57.4) |

List of abbreviations: NT-proBNP: N-terminal pro-B-type natriuretic peptide; ng: nanograms; hsTnT: high sensitive troponin T; hs-CRP: high-sensitive C-reactive protein; GDF15: Growth/differentiation factor 15; GAL-3: galectin-3.

**Supplementary Table S2: Added prognostic value in secondary outcomes of a multi-biomarker multi-point-based risk stratification strategy in acute heart failure**

|  | **C-index** | **P value**  **vs clinical model** | **G-B p-value** | **Brier score** | **AIC** | **BIC** | **IDI** | **P value**  **for IDI** | **NRI** | **P value**  **for NRI** |
| --- | --- | --- | --- | --- | --- | --- | --- | --- | --- | --- |
| **12-month CV mortality** | | | | | | | | | | |
| Clinical model (CM) | 0.770 (0.729-0.811) |  | 0.104 | 0.114 | 1456 | 1459 |  |  |  |  |
| CM + All biomarker | 0.780 (0.74-0.821) | <0.001 | 0.260 | 0.110 | 1432 | 1443 | 0.028 | 0.002 | 0.386 | <0.001 |
| CM + NT-proBNP, hs-CRP, GDF-15 | 0.786 (0.747-0.825) | <0.001 | <0.001 | 0.109 | 1437 | 1464 | 0.038 | <0.001 | 0.416 | <0.001 |
| **12-month HF mortality** | | | | | | | | | | |
| Clinical model (CM) | 0.780 (0.737-0.823) |  | 0.078 | 0.103 | 1280 | 1282 |  |  |  |  |
| CM + All biomarker | 0.793 (0.75-0.836) | <0.001 | 0.005 | 0.098 | 1242 | 1253 | 0.043 | 0.001 | 0.418 | <0.001 |
| CM + NT-proBNP, hs-CRP, GDF-15 | 0.797 (0.756-0.839) | <0.001 | <0.001 | 0.096 | 1251 | 1277 | 0.050 | <0.001 | 0.462 | <0.001 |
| **12-m HF admission** | | | | | | | | | | |
| Clinical model (CM) | 0.652 (0.618-0.685) |  | 0.770 | 0.226 | 3389 | 3393 |  |  |  |  |
| CM + All biomarker | 0.663 (0.63-0.696) | <0.001 | 0.890 | 0.223 | 3379 | 3393 | 0.009 | 0.039 | 0.157 | 0.035 |
| CM + NT-proBNP, hs-CRP, GDF-15 | 0.687 (0.656-0.719) | <0.001 | 0.254 | 0.221 | 3365 | 3401 | 0.036 | <0.001 | 0.331 | <0.001 |

List of abbreviations: G-B: Grønnesby and Borgan; AIC: Akaike criteria; BIC: Bayesian criteria; IDI: integrated discrimination improvement; NRI: net reclassification improvement; NT-proBNP: N-terminal pro-B-type natriuretic peptide; hs-CRP: high-sensitive C-reactive protein; GDF15: Growth/differentiation factor 15; CV: cardiovascular; HF: heart failure.

**Table S3: Added prognostic value of all multi-biomarker multi-point-based risk stratification strategies in acute heart failure**

|  | **C-index** | **P value**  **vs clinical model** | **G-B p-value** | **Brier score** | **AIC** | **BIC** | **IDI** | **P value**  **for IDI** | **NRI** | **P value**  **for NRI** |
| --- | --- | --- | --- | --- | --- | --- | --- | --- | --- | --- |
| **12-month all-cause mortality** | | | | | | | | | | |
| Clinical model (CM) | 0.752 (0.715-0.789) |  | 0.742 | 0.138 | 1908 | 1911 |  |  |  |  |
| CM + NT-proBNP, hs-CRP, GDF-15  *At Discharge | 0.767 (0.730-0.804) | <0.001 | 0.875 | 0.134 | 1892 | 1904 | 0.027 | <0.001 | 0.385 | <0.001 |
| CM + NT-proBNP, hs-CRP, GDF-15  *Admission / Discharge / Delta | 0.782 (0.747-0.817) | <0.001 | 0.550 | 0.133 | 1889 | 1919 | 0.050 | <0.001 | 0.566 | <0.001 |
| CM + NT-proBNP  *Admission / Discharge / Delta | 0.771 (0.735-0.807) | <0.001 | 0.972 | 0.135 | 1889 | 1901 | 0.030 | <0.001 | 0.478 | <0.001 |
| CM + NT-proBNP **VS**  CM + NT-proBNP, hs-CRP  *Admission / Discharge / Delta | 0.777 (0.743-0.812) | 0.218 | 0.526 | 0.134 | 1890 | 1912 | 0.004 | 0.243 | 0.142 | 0.107 |
| CM + NT-proBNP, hs-CRP **VS**  CM + NT-proBNP, hs-CRP, GDF-15  *Admission / Discharge / Delta | 0.782 (0.747-0.817) | 0.049 | 0.550 | 0.133 | 1889 | 1919 | 0.015 | 0.001 | 0.223 | 0.012 |
| **12-month all-cause mortality: 5 biomarkers 6 combinations** | | | | | | | | | | |
| Clinical model (CM) | 0.752 (0.715-0.789) |  | 0.742 | 0.138 | 1908 | 1911 |  |  |  |  |
| CM + All biomarker: except NT-proBNP | 0.770 (0.733-0.806) | 0.007 | 0.999 | 0.133 | 1906 | 1955 | 0.039 | <0.001 | 0.590 | <0.001 |
| CM + All biomarker: except Hs-TnT | 0.781 (0.746-0.817) | <0.001 | 0.999 | 0.133 | 1898 | 1947 | 0.051 | <0.001 | 0.587 | <0.001 |
| CM + All biomarker: except Cystatin C | 0.782 (0.748-0.817) | <0.001 | 0.190 | 0.132 | 1896 | 1945 | 0.054 | <0.001 | 0.538 | <0.001 |
| CM + All biomarker: except Hs-CRP | 0.780 (0.745-0.816) | <0.001 | 0.988 | 0.132 | 1897 | 1945 | 0.053 | <0.001 | 0.580 | <0.001 |
| CM + All biomarker: except GDF-15 | 0.778 (0.742-0.813) | <0.001 | 0.533 | 0.133 | 1900 | 1948 | 0.044 | <0.001 | 0.567 | <0.001 |
| CM + All biomarker: except GAL-3 | 0.783 (0.748-0.818) | <0.001 | 0.988 | 0.133 | 1895 | 1944 | 0.054 | <0.001 | 0.572 | <0.001 |
| **12-month CV mortality: 5 biomarkers 6 combinations** | | | | | | | | | | |
| Clinical model (CM) | 0.770 (0.729-0.811) |  | 0.104 | 0.114 | 1456 | 1459 |  |  |  |  |
| CM + All biomarker: except NT-proBNP | 0.785 (0.745-0.826) | <0.001 | 0.004 | 0.109 | 1447 | 1491 | 0.039 | <0.001 | 0.408 | <0.001 |
| CM + All biomarker: except Hs-TnT | 0.790 (0.750-0.830) | <0.001 | 0.017 | 0.108 | 1443 | 1487 | 0.045 | <0.001 | 0.515 | <0.001 |
| CM + All biomarker: except Cystatin C | 0.790 (0.752-0.828) | <0.001 | 0.001 | 0.108 | 1443 | 1488 | 0.044 | <0.001 | 0.529 | <0.001 |
| CM + All biomarker: except Hs-CRP | 0.789 (0.749-0.830) | <0.001 | 0.126 | 0.108 | 1444 | 1489 | 0.042 | <0.001 | 0.549 | <0.001 |
| CM + All biomarker: except GDF-15 | 0.791 (0.752-0.829) | <0.001 | 0.001 | 0.109 | 1445 | 1489 | 0.040 | <0.001 | 0.529 | <0.001 |
| CM + All biomarker: except GAL-3 | 0.791 (0.751-0.830) | <0.001 | 0.001 | 0.108 | 1442 | 1486 | 0.044 | <0.001 | 0.510 | <0.001 |
| **12-month HF mortality: 5 biomarkers 6 combinations** | | | | | | | | | | |
| Clinical model (CM) | 0.780 (0.737-0.823) |  | 0.078 | 0.103 | 1280 | 1282 |  |  |  |  |
| CM + All biomarker: except NT-proBNP | 0.796 (0.752-0.839) | <0.001 | 0.473 | 0.098 | 1265 | 1307 | 0.047 | <0.001 | 0.542 | <0.001 |
| CM + All biomarker: except Hs-TnT | 0.803 (0.761-0.845) | <0.001 | 0.001 | 0.096 | 1254 | 1296 | 0.061 | <0.001 | 0.506 | <0.001 |
| CM + All biomarker: except Cystatin C | 0.804 (0.764-0.845) | <0.001 | 0.001 | 0.096 | 1255 | 1298 | 0.055 | <0.001 | 0.548 | <0.001 |
| CM + All biomarker: except Hs-CRP | 0.801 (0.758-0.844) | <0.001 | 0.088 | 0.096 | 1258 | 1300 | 0.054 | <0.001 | 0.611 | <0.001 |
| CM + All biomarker: except GDF-15 | 0.805 (0.764-0.845) | <0.001 | 0.001 | 0.097 | 1258 | 1301 | 0.052 | <0.001 | 0.567 | <0.001 |
| CM + All biomarker: except GAL-3 | 0.807 (0.766-0.849) | <0.001 | 0.848 | 0.096 | 1254 | 1296 | 0.060 | <0.001 | 0.649 | <0.001 |
| **12-m HF admission: 5 biomarkers 6 combinations** | | | | | | | | | | |
| Clinical model (CM) | 0.652 (0.618-0.685) |  | 0.770 | 0.226 | 3389 | 3393 |  |  |  |  |
| CM + All biomarker: except NT-proBNP | 0.685 (0.654-0.717) | <0.001 | 0.923 | 0.219 | 3372 | 3430 | 0.047 | <0.001 | 0.418 | <0.001 |
| CM + All biomarker: except Hs-TnT | 0.697 (0.666-0.728) | <0.001 | 0.675 | 0.219 | 3363 | 3421 | 0.058 | <0.001 | 0.453 | <0.001 |
| CM + All biomarker: except Cystatin C | 0.694 (0.664-0.725) | <0.001 | 1.000 | 0.218 | 3362 | 3419 | 0.057 | <0.001 | 0.456 | <0.001 |
| CM + All biomarker: except Hs-CRP | 0.701 (0.67-0.732) | <0.001 | 0.133 | 0.218 | 3357 | 3414 | 0.064 | <0.001 | 0.460 | <0.001 |
| CM + All biomarker: except GDF-15 | 0.694 (0.664-0.725) | <0.001 | 0.951 | 0.218 | 3360 | 3417 | 0.058 | <0.001 | 0.493 | <0.001 |
| CM + All biomarker: except GAL-3 | 0.697 (0.667-0.728) | <0.001 | 0.867 | 0.218 | 3358 | 3416 | 0.059 | <0.001 | 0.477 | <0.001 |
| **12-month all-cause mortality: 4 biomarkers 15 combinations** | | | | | | | | | | |
| Clinical model (CM) | 0.752 (0.715-0.789) |  | 0.742 | 0.138 | 1908 | 1911 |  |  |  |  |
| CM + (NT-PROBNP, Hs-TnT, Cystatin C, HS-CRP) | 0.778 (0.742-0.813) | <0.001 | 0.430 | 0.133 | 1895 | 1934 | 0.043 | <0.001 | 0.576 | <0.001 |
| CM + (NT-PROBNP, Hs-TnT, Cystatin C, GAL-3) | 0.773 (0.737-0.810) | <0.001 | 0.805 | 0.133 | 1897 | 1936 | 0.041 | <0.001 | 0.491 | <0.001 |
| CM + (NT-PROBNP, Hs-TnT, Cystatin C, GDF-15) | 0.780 (0.745-0.816) | <0.001 | 0.928 | 0.133 | 1892 | 1932 | 0.052 | <0.001 | 0.558 | <0.001 |
| CM + (NT-PROBNP, Hs-TnT, HS-CRP, GAL-3) | 0.777 (0.742-0.811) | <0.001 | 0.172 | 0.133 | 1896 | 1936 | 0.041 | <0.001 | 0.556 | <0.001 |
| CM + (NT-PROBNP, Hs-TnT, HS-CRP, GDF-15) | 0.783 (0.748-0.818) | <0.001 | 0.953 | 0.132 | 1891 | 1930 | 0.054 | <0.001 | 0.524 | <0.001 |
| CM + (NT-PROBNP, Hs-TnT, GAL-3, GDF-15) | 0.779 (0.744-0.815) | <0.001 | 0.974 | 0.132 | 1893 | 1933 | 0.052 | <0.001 | 0.557 | <0.001 |
| CM + (NT-PROBNP, Cystatin C, HS-CRP, GAL-3) | 0.776 (0.740-0.812) | <0.001 | 0.860 | 0.134 | 1897 | 1937 | 0.040 | <0.001 | 0.479 | <0.001 |
| CM + (NT-PROBNP, Cystatin C, HS-CRP, GDF-15) | 0.781 (0.746-0.817) | <0.001 | 0.999 | 0.133 | 1893 | 1932 | 0.050 | <0.001 | 0.527 | <0.001 |
| CM + (NT-PROBNP, Cystatin C, GAL-3, GDF-15) | 0.778 (0.741-0.814) | <0.001 | 1 | 0.133 | 1895 | 1934 | 0.049 | <0.001 | 0.602 | <0.001 |
| CM + (NT-PROBNP, HS-CRP, GAL-3, GDF-15) | 0.781 (0.746-0.816) | <0.001 | 0.493 | 0.133 | 1894 | 1933 | 0.050 | <0.001 | 0.575 | <0.001 |
| CM + (Hs-TnT, Cystatin C, HS-CRP, GAL-3) | 0.759 (0.722-0.797) | 0.035 | 0.978 | 0.135 | 1910 | 1949 | 0.023 | 0.001 | 0.260 | 0.003 |
| CM + (Hs-TnT, Cystatin C, HS-CRP, GDF-15) | 0.769 (0.733-0.806) | 0.002 | 0.727 | 0.133 | 1902 | 1941 | 0.038 | <0.001 | 0.512 | <0.001 |
| CM + (Hs-TnT, Cystatin C, GAL-3, GDF-15) | 0.766 (0.729-0.803) | 0.004 | 0.999 | 0.133 | 1903 | 1943 | 0.036 | <0.001 | 0.427 | <0.001 |
| CM + (Hs-TnT, HS-CRP, GAL-3, GDF-15) | 0.769 (0.733-0.806) | 0.003 | 0.992 | 0.133 | 1902 | 1942 | 0.038 | <0.001 | 0.487 | <0.001 |
| CM + (Cystatin C, HS-CRP, GAL-3, GDF-15) | 0.766 (0.728-0.803) | 0.009 | 0.120 | 0.134 | 1906 | 1945 | 0.034 | <0.001 | 0.508 | <0.001 |
| **12-month CV mortality: 4 biomarkers 15 combinations** | | | | | | | | | | |
| Clinical model (CM) | 0.770 (0.729-0.811) |  | 0.104 | 0.114 | 1456 | 1459 |  |  |  |  |
| CM + (NT-PROBNP, Hs-TnT, Cystatin C, HS-CRP) | 0.788 (0.748-0.827) | <0.001 | 0.001 | 0.109 | 1442 | 1478 | 0.037 | <0.001 | 0.445 | <0.001 |
| CM + (NT-PROBNP, Hs-TnT, Cystatin C, GAL-3) | 0.784 (0.743-0.824) | <0.001 | 0.744 | 0.109 | 1444 | 1480 | 0.034 | <0.001 | 0.473 | <0.001 |
| CM + (NT-PROBNP, Hs-TnT, Cystatin C, GDF-15) | 0.786 (0.745-0.827) | <0.001 | 0.343 | 0.109 | 1442 | 1478 | 0.038 | <0.001 | 0.479 | <0.001 |
| CM + (NT-PROBNP, Hs-TnT, HS-CRP, GAL-3) | 0.788 (0.75-0.826) | <0.001 | 0.001 | 0.109 | 1443 | 1479 | 0.037 | <0.001 | 0.484 | <0.001 |
| CM + (NT-PROBNP, Hs-TnT, HS-CRP, GDF-15) | 0.789 (0.751-0.828) | <0.001 | 0.001 | 0.109 | 1439 | 1475 | 0.041 | <0.001 | 0.521 | <0.001 |
| CM + (NT-PROBNP, Hs-TnT, GAL-3, GDF-15) | 0.785 (0.745-0.824) | <0.001 | 0.632 | 0.109 | 1443 | 1479 | 0.037 | <0.001 | 0.620 | <0.001 |
| CM + (NT-PROBNP, Cystatin C, HS-CRP, GAL-3) | 0.787 (0.748 -0.827) | <0.001 | 0.001 | 0.109 | 1443 | 1479 | 0.037 | <0.001 | 0.397 | <0.001 |
| CM + (NT-PROBNP, Cystatin C, HS-CRP, GDF-15) | 0.788 (0.748-0.828) | <0.001 | 0.017 | 0.108 | 1440 | 1476 | 0.042 | <0.001 | 0.402 | <0.001 |
| CM + (NT-PROBNP, Cystatin C, GAL-3, GDF-15) | 0.789 (0.748-0.830) | <0.001 | 0.948 | 0.109 | 1443 | 1479 | 0.038 | <0.001 | 0.532 | <0.001 |
| CM + (NT-PROBNP, HS-CRP, GAL-3, GDF-15) | 0.787 (0.748-0.825) | <0.001 | 0.001 | 0.108 | 1441 | 1477 | 0.041 | <0.001 | 0.453 | <0.001 |
| CM + (Hs-TnT, Cystatin C, HS-CRP, GAL-3) | 0.782 (0.743-0.822) | 0.002 | 0.002 | 0.111 | 1450 | 1486 | 0.029 | 0.002 | 0.420 | <0.001 |
| CM + (Hs-TnT, Cystatin C, HS-CRP, GDF-15 ) | 0.781 (0.740-0.823) | <0.001 | 0.008 | 0.109 | 1444 | 1480 | 0.035 | <0.001 | 0.513 | <0.001 |
| CM + (Hs-TnT, Cystatin C, GAL-3, GDF-15) | 0.781 (0.740-0.822) | <0.001 | 0.673 | 0.109 | 1446 | 1482 | 0.032 | <0.001 | 0.448 | <0.001 |
| CM + (Hs-TnT, HS-CRP, GAL-3, GDF-15) | 0.781 (0.741-0.821) | <0.001 | 0.001 | 0.109 | 1445 | 1481 | 0.036 | <0.001 | 0.439 | <0.001 |
| CM + (Cystatin C, HS-CRP, GAL-3, GDF-15) | 0.781 (0.739-0.823) | <0.001 | 0.042 | 0.109 | 1447 | 1483 | 0.034 | <0.001 | 0.402 | <0.001 |
| **12-month HF mortality: 4 biomarkers 15 combinations** | | | | | | | | | | |
| Clinical model (CM) | 0.780 (0.737-0.823) |  | 0.078 | 0.103 | 1280 | 1282 |  |  |  |  |
| CM + (NT-PROBNP, Hs-TnT, Cystatin C, HS-CRP) | 0.804 (0.762-0.846) | <0.001 | 0.001 | 0.097 | 1255 | 1289 | 0.050 | <0.001 | 0.550 | <0.001 |
| CM + (NT-PROBNP, Hs-TnT, Cystatin C, GAL-3) | 0.797 (0.754-0.841) | <0.001 | 0.999 | 0.098 | 1258 | 1293 | 0.043 | <0.001 | 0.504 | <0.001 |
| CM + (NT-PROBNP, Hs-TnT, Cystatin C, GDF-15) | 0.801 (0.757-0.844) | <0.001 | 0.429 | 0.097 | 1254 | 1289 | 0.051 | <0.001 | 0.589 | <0.001 |
| CM + (NT-PROBNP, Hs-TnT, HS-CRP, GAL-3) | 0.802 (0.762-0.842) | <0.001 | 0.001 | 0.097 | 1255 | 1290 | 0.046 | <0.001 | 0.506 | <0.001 |
| CM + (NT-PROBNP, Hs-TnT, HS-CRP, GDF-15) | 0.802 (0.762-0.842) | <0.001 | 0.001 | 0.097 | 1253 | 1288 | 0.052 | <0.001 | 0.589 | <0.001 |
| CM + (NT-PROBNP, Hs-TnT, GAL-3, GDF-15) | 0.799 (0.758-0.841) | <0.001 | 0.217 | 0.097 | 1256 | 1290 | 0.045 | <0.001 | 0.611 | <0.001 |
| CM + (NT-PROBNP, Cystatin C, HS-CRP, GAL-3) | 0.800 (0.758-0.841) | <0.001 | 0.001 | 0.097 | 1255 | 1290 | 0.050 | <0.001 | 0.407 | <0.001 |
| CM + (NT-PROBNP, Cystatin C, HS-CRP, GDF-15) | 0.803 (0.760-0.845) | <0.001 | 0.014 | 0.096 | 1251 | 1285 | 0.058 | <0.001 | 0.506 | <0.001 |
| CM + (NT-PROBNP, Cystatin C, GAL-3, GDF-15) | 0.799 (0.755-0.844) | <0.001 | 0.998 | 0.097 | 1256 | 1290 | 0.050 | <0.001 | 0.479 | <0.001 |
| CM + (NT-PROBNP, HS-CRP, GAL-3, GDF-15) | 0.798 (0.757-0.839) | <0.001 | 0.001 | 0.096 | 1253 | 1287 | 0.053 | <0.001 | 0.495 | <0.001 |
| CM + (Hs-TnT, Cystatin C, HS-CRP, GAL-3) | 0.789 (0.746-0.833) | 0.001 | 0.654 | 0.100 | 1271 | 1305 | 0.033 | 0.006 | 0.396 | <0.001 |
| CM + (Hs-TnT, Cystatin C, HS-CRP, GDF-15) | 0.796 (0.753-0.840) | <0.001 | 0.554 | 0.098 | 1260 | 1294 | 0.045 | <0.001 | 0.594 | <0.001 |
| CM + (Hs-TnT, Cystatin C, GAL-3, GDF-15) | 0.786 (0.740-0.832) | <0.001 | 0.997 | 0.098 | 1266 | 1300 | 0.038 | <0.001 | 0.471 | <0.001 |
| CM + (Hs-TnT, HS-CRP, GAL-3, GDF-15) | 0.790 (0.746-0.834) | <0.001 | 0.999 | 0.098 | 1263 | 1297 | 0.041 | <0.001 | 0.564 | <0.001 |
| CM + (Cystatin C, HS-CRP, GAL-3, GDF-15) | 0.788 (0.741-0.834) | <0.001 | 0.991 | 0.098 | 1266 | 1300 | 0.042 | <0.001 | 0.454 | <0.001 |
| **12-m HF admission: 4 biomarkers 15 combinations** | | | | | | | | | | |
| Clinical model (CM) | 0.652 (0.618-0.685) |  | 0.770 | 0.226 | 3389 | 3393 |  |  |  |  |
| CM + (NT-PROBNP, Hs-TnT, Cystatin C, HS-CRP) | 0.692 (0.661-0.722) | <0.001 | 0.610 | 0.218 | 3358 | 3404 | 0.052 | <0.001 | 0.608 | <0.001 |
| CM + (NT-PROBNP, Hs-TnT, Cystatin C, GAL-3) | 0.692 (0.661-0.723) | <0.001 | 0.449 | 0.218 | 3359 | 3405 | 0.053 | <0.001 | 0.470 | <0.001 |
| CM + (NT-PROBNP, Hs-TnT, Cystatin C, GDF-15) | 0.696 (0.665-0.726) | <0.001 | 0.200 | 0.219 | 3356 | 3403 | 0.056 | <0.001 | 0.400 | <0.001 |
| CM + (NT-PROBNP, Hs-TnT, HS-CRP, GAL-3) | 0.690 (0.659-0.721) | <0.001 | 0.951 | 0.218 | 3360 | 3407 | 0.051 | <0.001 | 0.455 | <0.001 |
| CM + (NT-PROBNP, Hs-TnT, HS-CRP, GDF-15) | 0.687 (0.657-0.718) | <0.001 | 0.734 | 0.220 | 3366 | 3413 | 0.042 | <0.001 | 0.312 | <0.001 |
| CM + (NT-PROBNP, Hs-TnT, GAL-3, GDF-15) | 0.695 (0.664-0.726) | <0.001 | 0.682 | 0.219 | 3361 | 3408 | 0.052 | <0.001 | 0.456 | <0.001 |
| CM + (NT-PROBNP, Cystatin C, HS-CRP, GAL-3) | 0.690 (0.659-0.721) | <0.001 | 0.169 | 0.220 | 3366 | 3413 | 0.046 | <0.001 | 0.426 | <0.001 |
| CM + (NT-PROBNP, Cystatin C, HS-CRP, GDF-15) | 0.693 (0.662-0.724) | <0.001 | 0.038 | 0.220 | 3362 | 3408 | 0.050 | <0.001 | 0.396 | <0.001 |
| CM + (NT-PROBNP, Cystatin C, GAL-3, GDF-15) | 0.699 (0.668-0.730) | <0.001 | 0.197 | 0.220 | 3363 | 3409 | 0.053 | <0.001 | 0.386 | <0.001 |
| CM + (NT-PROBNP, HS-CRP, GAL-3, GDF-15) | 0.692 (0.661-0.723) | <0.001 | 0.117 | 0.220 | 3365 | 3412 | 0.049 | <0.001 | 0.359 | <0.001 |
| CM + (Hs-TnT, Cystatin C, HS-CRP, GAL-3) | 0.677 (0.646-0.708) | <0.001 | 0.634 | 0.220 | 3375 | 3422 | 0.037 | <0.001 | 0.299 | <0.001 |
| CM + (Hs-TnT, Cystatin C, HS-CRP, GDF-15) | 0.680 (0.649-0.712) | <0.001 | 0.306 | 0.220 | 3372 | 3419 | 0.039 | <0.001 | 0.380 | <0.001 |
| CM + (Hs-TnT, Cystatin C, GAL-3, GDF-15) | 0.686 (0.654-0.718) | <0.001 | 0.700 | 0.220 | 3371 | 3418 | 0.043 | <0.001 | 0.369 | <0.001 |
| CM + (Hs-TnT, HS-CRP, GAL-3, GDF-15) | 0.679 (0.648-0.710) | <0.001 | 0.999 | 0.220 | 3375 | 3422 | 0.036 | <0.001 | 0.275 | <0.001 |
| CM + (Cystatin C, HS-CRP, GAL-3, GDF-15) | 0.680 (0.648-0.712) | <0.001 | 0.454 | 0.221 | 3378 | 3425 | 0.035 | <0.001 | 0.391 | <0.001 |
| **12-month all-cause mortality: 3 biomarkers 20 combinations** | | | | | | | | | | |
| Clinical model (CM) | 0.752 (0.715-0.789) |  | 0.742 | 0.138 | 1908 | 1911 |  |  |  |  |
| CM + (NT-PROBNP, Hs-TnT, Cystatin C) | 0.773 (0.737-0.809) | <0.001 | 0.993 | 0.134 | 1892 | 1923 | 0.040 | <0.001 | 0.533 | <0.001 |
| CM + (NT-PROBNP, Hs-TnT, HS-CRP) | 0.777 (0.742-0.812) | <0.001 | 0.581 | 0.134 | 1892 | 1922 | 0.039 | <0.001 | 0.484 | <0.001 |
| CM + (NT-PROBNP, Hs-TnT, GAL-3) | 0.771 (0.735-0.807) | <0.001 | 0.937 | 0.134 | 1894 | 1925 | 0.037 | <0.001 | 0.562 | <0.001 |
| CM + (NT-PROBNP, Hs-TnT, GDF-15) | 0.780 (0.744-0.815) | <0.001 | 0.581 | 0.133 | 1888 | 1919 | 0.052 | <0.001 | 0.547 | <0.001 |
| CM + (NT-PROBNP, Cystatin C, HS-CRP) | 0.776 (0.740-0.811) | <0.001 | 0.999 | 0.134 | 1893 | 1923 | 0.039 | <0.001 | 0.450 | <0.001 |
| CM + (NT-PROBNP, Cystatin C, GAL-3) | 0.772 (0.735-0.809) | <0.001 | 1 | 0.134 | 1895 | 1925 | 0.037 | <0.001 | 0.537 | <0.001 |
| CM + (NT-PROBNP, Cystatin C, GDF-15) | 0.778 (0.741-0.814) | <0.001 | 0.996 | 0.133 | 1890 | 1920 | 0.048 | <0.001 | 0.528 | <0.001 |
| CM + (NT-PROBNP, HS-CRP GAL-3) | 0.776 (0.741-0.810) | <0.001 | 0.808 | 0.134 | 1894 | 1925 | 0.036 | <0.001 | 0.426 | <0.001 |
| CM + (NT-PROBNP, HS-CRP, GDF-15) | 0.782 (0.747-0.817) | <0.001 | 0.550 | 0.133 | 1889 | 1919 | 0.050 | <0.001 | 0.566 | <0.001 |
| CM + (NT-PROBNP, GAL-3, GDF-15) | 0.777 (0.741-0.813) | <0.001 | 0.623 | 0.133 | 1891 | 1921 | 0.047 | <0.001 | 0.527 | <0.001 |
| CM + (Hs-TnT, Cystatin C, HS-CRP) | 0.759 (0.722-0.796) | 0.015 | 0.937 | 0.135 | 1906 | 1936 | 0.021 | 0.002 | 0.250 | 0.005 |
| CM + (Hs-TnT, Cystatin C, GAL-3) | 0.753 (0.715-0.791) | 0.035 | 0.577 | 0.135 | 1908 | 1939 | 0.019 | 0.002 | 0.283 | 0.001 |
| CM + (Hs-TnT, Cystatin C, GDF-15) | 0.766 (0.729-0.803) | 0.001 | 0.999 | 0.134 | 1899 | 1929 | 0.035 | <0.001 | 0.439 | <0.001 |
| CM + (Hs-TnT, HS-CRP, GAL-3) | 0.758 (0.720-0.795) | 0.031 | 1 | 0.135 | 1908 | 1938 | 0.019 | 0.003 | 0.308 | <0.001 |
| CM + (Hs-TnT, HS-CRP, GDF-15) | 0.769 (0.732-0.806) | <0.001 | 0.345 | 0.133 | 1897 | 1928 | 0.038 | <0.001 | 0.497 | <0.001 |
| CM + (Hs-TnT, GAL-3, GDF-15) | 0.765 (0.728-0.803) | 0.002 | 1 | 0.133 | 1900 | 1930 | 0.035 | <0.001 | 0.385 | <0.001 |
| CM + (Cystatin C, HS-CRP, GAL-3) | 0.758 (0.721-0.795) | 0.064 | 0.620 | 0.136 | 1910 | 1941 | 0.016 | 0.006 | 0.245 | 0.006 |
| CM + (Cystatin C, HS-CRP, GDF-15) | 0.765 (0.728-0.803) | 0.003 | 0.225 | 0.134 | 1901 | 1931 | 0.033 | <0.001 | 0.406 | <0.001 |
| CM + (Cystatin C, GAL-3, GDF-15) | 0.763 (0.725-0.801) | 0.005 | 0.937 | 0.134 | 1903 | 1933 | 0.030 | <0.001 | 0.404 | <0.001 |
| CM + (HS-CRP, GAL-3, GDF-15) | 0.765 (0.727-0.803) | 0.004 | 0.995 | 0.134 | 1902 | 1932 | 0.033 | <0.001 | 0.550 | <0.001 |
| **12-month CV mortality: 3 biomarkers 20 combinations** | | | | | | | | | | |
| Clinical model (CM) | 0.770 (0.729-0.811) |  | 0.104 | 0.114 | 1456 | 1459 |  |  |  |  |
| CM + (NT-PROBNP, Hs-TnT, Cystatin C) | 0.780 (0.739-0.822) | <0.001 | 0.537 | 0.110 | 1442 | 1469 | 0.030 | <0.001 | 0.428 | <0.001 |
| CM + (NT-PROBNP, Hs-TnT, HS-CRP) | 0.787 (0.748-0.825) | <0.001 | 0.001 | 0.110 | 1438 | 1466 | 0.034 | <0.001 | 0.490 | <0.001 |
| CM + (NT-PROBNP, Hs-TnT, GAL-3) | 0.779 (0.739-0.819) | <0.001 | 0.382 | 0.110 | 1443 | 1470 | 0.030 | <0.001 | 0.507 | <0.001 |
| CM + (NT-PROBNP, Hs-TnT, GDF-15 ) | 0.784 (0.744-0.824) | <0.001 | 0.552 | 0.109 | 1439 | 1467 | 0.035 | <0.001 | 0.583 | <0.001 |
| CM + (NT-PROBNP, Cystatin C, HS-CRP) | 0.784 (0.744-0.824) | <0.001 | 0.001 | 0.110 | 1440 | 1467 | 0.033 | 0.001 | 0.451 | <0.001 |
| CM + (NT-PROBNP, Cystatin C, GAL-3) | 0.783 (0.741-0.824) | <0.001 | 0.987 | 0.110 | 1443 | 1471 | 0.030 | <0.001 | 0.425 | <0.001 |
| CM + (NT-PROBNP, Cystatin C, GDF-15) | 0.785 (0.744-0.827) | <0.001 | 0.977 | 0.109 | 1441 | 1468 | 0.034 | <0.001 | 0.411 | <0.001 |
| CM + (NT-PROBNP, HS-CRP, GAL-3) | 0.785 (0.747-0.823) | <0.001 | 0.001 | 0.110 | 1442 | 1470 | 0.032 | <0.001 | 0.422 | <0.001 |
| CM + (NT-PROBNP, HS-CRP, GDF-15) | 0.786 (0.747-0.825) | <0.001 | 0.001 | 0.109 | 1437 | 1464 | 0.038 | <0.001 | 0.416 | <0.001 |
| CM + (NT-PROBNP, GAL-3, GDF-15) | 0.784 (0.745-0.824) | <0.001 | 0.272 | 0.110 | 1442 | 1470 | 0.033 | <0.001 | 0.453 | <0.001 |
| CM + (Hs-TnT, Cystatin C, HS-CRP) | 0.777 (0.737-0.818) | 0.002 | 0.159 | 0.111 | 1447 | 1475 | 0.025 | 0.005 | 0.417 | <0.001 |
| CM + (Hs-TnT, Cystatin C, GAL-3) | 0.774 (0.733-0.816) | 0.005 | 0.335 | 0.111 | 1450 | 1478 | 0.021 | 0.005 | 0.349 | <0.001 |
| CM + (Hs-TnT, Cystatin C, GDF-15) | 0.776 (0.734-0.818) | <0.001 | 0.361 | 0.110 | 1445 | 1473 | 0.028 | <0.001 | 0.375 | <0.001 |
| CM + (Hs-TnT, HS-CRP, GAL-3) | 0.776 (0.736-0.816) | 0.002 | 1 | 0.111 | 1448 | 1476 | 0.026 | 0.002 | 0.383 | <0.001 |
| CM + (Hs-TnT, HS-CRP, GDF-15) | 0.780 (0.739-0.820) | <0.001 | 0.001 | 0.109 | 1441 | 1468 | 0.033 | <0.001 | 0.442 | <0.001 |
| CM + (Hs-TnT, GAL-3, GDF-15) | 0.775 (0.735-0.816) | <0.001 | 0.856 | 0.110 | 1445 | 1473 | 0.029 | <0.001 | 0.400 | <0.001 |
| CM + (Cystatin C, HS-CRP, GAL-3) | 0.777 (0.737-0.817) | 0.005 | 0.671 | 0.111 | 1451 | 1478 | 0.021 | 0.009 | 0.343 | <0.001 |
| CM + (Cystatin C, HS-CRP, GDF-15) | 0.775 (0.732-0.818) | <0.001 | 0.486 | 0.110 | 1445 | 1472 | 0.030 | <0.001 | 0.388 | <0.001 |
| CM + (Cystatin C, GAL-3, GDF-15) | 0.778 (0.737-0.820) | 0.002 | 0.247 | 0.110 | 1447 | 1475 | 0.025 | <0.001 | 0.417 | <0.001 |
| CM + (HS-CRP, GAL-3, GDF-15) | 0.775 (0.733-0.816) | <0.001 | 0.239 | 0.110 | 1446 | 1474 | 0.031 | <0.001 | 0.335 | <0.001 |
| **12-month HF mortality: 3 biomarkers 20 combinations** | | | | | | | | | | |
| Clinical model (CM) | 0.780 (0.737-0.823) |  | 0.078 | 0.103 | 1280 | 1282 |  |  |  |  |
| CM + (NT-PROBNP, Hs-TnT, Cystatin C) | 0.797 (0.752-0.842) | <0.001 | 0.892 | 0.098 | 1255 | 1282 | 0.041 | <0.001 | 0.567 | <0.001 |
| CM + (NT-PROBNP, Hs-TnT, HS-CRP) | 0.800 (0.760-0.841) | <0.001 | 0.001 | 0.098 | 1254 | 1280 | 0.043 | <0.001 | 0.561 | <0.001 |
| CM + (NT-PROBNP, Hs-TnT, GAL-3) | 0.795 (0.754-0.837) | <0.001 | 0.441 | 0.098 | 1255 | 1282 | 0.037 | 0.001 | 0.476 | <0.001 |
| CM + (NT-PROBNP, Hs-TnT, GDF-15) | 0.796 (0.753-0.838) | <0.001 | 0.608 | 0.097 | 1255 | 1281 | 0.043 | <0.001 | 0.534 | <0.001 |
| CM + (NT-PROBNP, Cystatin C, HS-CRP) | 0.799 (0.757-0.842) | <0.001 | 0.001 | 0.097 | 1252 | 1278 | 0.048 | <0.001 | 0.454 | <0.001 |
| CM + (NT-PROBNP, Cystatin C, GAL-3) | 0.795 (0.751-0.84) | <0.001 | 0.835 | 0.098 | 1257 | 1283 | 0.039 | <0.001 | 0.476 | <0.001 |
| CM + (NT-PROBNP, Cystatin C, GDF-15) | 0.798 (0.753-0.843) | <0.001 | 0.982 | 0.097 | 1252 | 1279 | 0.048 | <0.001 | 0.570 | <0.001 |
| CM + (NT-PROBNP, HS-CRP, GAL-3) | 0.798 (0.759-0.838) | <0.001 | 0.001 | 0.097 | 1254 | 1280 | 0.043 | 0.001 | 0.465 | <0.001 |
| CM + (NT-PROBNP, HS-CRP, GDF-15) | 0.797 (0.756-0.838) | <0.001 | 0.001 | 0.096 | 1251 | 1277 | 0.050 | <0.001 | 0.462 | <0.001 |
| CM + (NT-PROBNP, GAL-3, GDF-15) | 0.796 (0.754-0.839) | <0.001 | 0.978 | 0.097 | 1255 | 1281 | 0.042 | <0.001 | 0.520 | <0.001 |
| CM + (Hs-TnT, Cystatin C, HS-CRP) | 0.790 (0.747-0.834) | <0.001 | 0.922 | 0.100 | 1266 | 1293 | 0.031 | 0.006 | 0.498 | <0.001 |
| CM + (Hs-TnT, Cystatin C, GAL-3) | 0.776 (0.728-0.823) | 0.003 | 0.454 | 0.100 | 1273 | 1299 | 0.023 | 0.017 | 0.273 | 0.009 |
| CM + (Hs-TnT, Cystatin C, GDF-15) | 0.787 (0.741-0.833) | <0.001 | 0.594 | 0.098 | 1261 | 1288 | 0.036 | <0.001 | 0.454 | <0.001 |
| CM + (Hs-TnT, HS-CRP, GAL-3) | 0.785 (0.742-0.828) | <0.001 | 0.198 | 0.100 | 1269 | 1296 | 0.028 | 0.011 | 0.374 | <0.001 |
| CM + (Hs-TnT, HS-CRP, GDF-15) | 0.791 (0.748-0.834) | <0.001 | 0.523 | 0.098 | 1259 | 1286 | 0.037 | 0.001 | 0.487 | <0.001 |
| CM + (Hs-TnT, GAL-3, GDF-15) | 0.781 (0.736-0.826) | <0.001 | 0.128 | 0.099 | 1264 | 1290 | 0.031 | 0.002 | 0.418 | <0.001 |
| CM + (Cystatin C, HS-CRP, GAL-3) | 0.784 (0.739-0.828) | 0.003 | 0.141 | 0.100 | 1273 | 1299 | 0.024 | 0.022 | 0.322 | 0.002 |
| CM + (Cystatin C, HS-CRP, GDF-15) | 0.788 (0.742-0.834) | <0.001 | 0.999 | 0.098 | 1260 | 1287 | 0.039 | <0.001 | 0.413 | <0.001 |
| CM + (Cystatin C, GAL-3, GDF-15) | 0.781 (0.734-0.829) | <0.001 | 0.899 | 0.099 | 1268 | 1294 | 0.030 | 0.002 | 0.385 | <0.001 |
| CM + (HS-CRP, GAL-3, GDF-15) | 0.781 (0.735-0.828) | <0.001 | 0.779 | 0.098 | 1265 | 1291 | 0.035 | 0.001 | 0.341 | 0.001 |
| **12-m HF admission: 3 biomarkers 20 combinations** | | | | | | | | | | |
| Clinical model (CM) | 0.652 (0.618-0.685) |  | 0.770 | 0.226 | 3389 | 3393 |  |  |  |  |
| CM + (NT-PROBNP, Hs-TnT, Cystatin C) | 0.688 (0.657-0.719) | <0.001 | 0.068 | 0.219 | 3356 | 3392 | 0.048 | <0.001 | 0.425 | <0.001 |
| CM + (NT-PROBNP, Hs-TnT, HS-CRP) | 0.684 (0.654-0.714) | <0.001 | 0.732 | 0.220 | 3362 | 3398 | 0.039 | <0.001 | 0.400 | <0.001 |
| CM + (NT-PROBNP, Hs-TnT, GAL-3) | 0.688 (0.658-0.719) | <0.001 | 0.989 | 0.219 | 3360 | 3395 | 0.046 | <0.001 | 0.435 | <0.001 |
| CM + (NT-PROBNP, Hs-TnT, GDF-15) | 0.687 (0.656-0.718) | <0.001 | 0.072 | 0.220 | 3363 | 3398 | 0.039 | <0.001 | 0.349 | <0.001 |
| CM + (NT-PROBNP, Cystatin C, HS-CRP) | 0.687 (0.655-0.718) | <0.001 | 0.824 | 0.220 | 3364 | 3399 | 0.040 | <0.001 | 0.430 | <0.001 |
| CM + (NT-PROBNP, Cystatin C, GAL-3) | 0.688 (0.656-0.719) | <0.001 | 0.570 | 0.221 | 3367 | 3403 | 0.039 | <0.001 | 0.369 | <0.001 |
| CM + (NT-PROBNP, Cystatin C, GDF-15) | 0.694 (0.663-0.725) | <0.001 | 0.075 | 0.221 | 3361 | 3397 | 0.045 | <0.001 | 0.374 | <0.001 |
| CM + (NT-PROBNP, HS-CRP, GAL-3) | 0.686 (0.655-0.717) | <0.001 | 0.727 | 0.220 | 3365 | 3401 | 0.040 | <0.001 | 0.424 | <0.001 |
| CM + (NT-PROBNP, HS-CRP, GDF-15) | 0.687 (0.656-0.719) | <0.001 | 0.254 | 0.221 | 3365 | 3401 | 0.036 | <0.001 | 0.331 | <0.001 |
| CM + (NT-PROBNP, GAL-3, GDF-15) | 0.695 (0.664-0.726) | <0.001 | 0.495 | 0.221 | 3364 | 3400 | 0.043 | <0.001 | 0.373 | <0.001 |
| CM + (Hs-TnT, Cystatin C, HS-CRP) | 0.672 (0.641-0.704) | <0.001 | 0.149 | 0.221 | 3373 | 3409 | 0.031 | <0.001 | 0.352 | <0.001 |
| CM + (Hs-TnT, Cystatin C, GAL-3) | 0.674 (0.642-0.706) | <0.001 | 0.979 | 0.221 | 3376 | 3411 | 0.031 | <0.001 | 0.297 | <0.001 |
| CM + (Hs-TnT, Cystatin C, GDF-15) | 0.680 (0.649-0.712) | <0.001 | 0.605 | 0.221 | 3369 | 3405 | 0.036 | <0.001 | 0.395 | <0.001 |
| CM + (Hs-TnT, HS-CRP, GAL-3) | 0.671 (0.64-0.702) | <0.001 | 1.000 | 0.221 | 3376 | 3412 | 0.030 | <0.001 | 0.318 | <0.001 |
| CM + (Hs-TnT, HS-CRP, GDF-15) | 0.673 (0.641-0.704) | <0.001 | 0.480 | 0.222 | 3376 | 3412 | 0.024 | <0.001 | 0.320 | <0.001 |
| CM + (Hs-TnT, GAL-3, GDF-15) | 0.681 (0.649-0.712) | <0.001 | 0.918 | 0.221 | 3373 | 3409 | 0.032 | <0.001 | 0.331 | <0.001 |
| CM + (Cystatin C, HS-CRP, GAL-3) | 0.666 (0.634-0.699) | 0.006 | 0.012 | 0.222 | 3384 | 3420 | 0.022 | <0.001 | 0.233 | 0.002 |
| CM + (Cystatin C, HS-CRP, GDF-15) | 0.676 (0.644-0.708) | <0.001 | 0.016 | 0.222 | 3375 | 3411 | 0.029 | <0.001 | 0.401 | <0.001 |
| CM + (Cystatin C, GAL-3, GDF-15) | 0.681 (0.648-0.713) | <0.001 | 0.130 | 0.222 | 3377 | 3413 | 0.031 | <0.001 | 0.357 | <0.001 |
| CM + (HS-CRP, GAL-3, GDF-15) | 0.676 (0.645-0.708) | <0.001 | 0.926 | 0.222 | 3376 | 3412 | 0.028 | <0.001 | 0.264 | <0.001 |

List of abbreviations: G-B: Grønnesby and Borgan; AIC: Akaike criteria; BIC: Bayesian criteria; IDI: integrated discrimination improvement; NRI: net reclassification improvement; NT-proBNP: N-terminal pro-B-type natriuretic peptide; hs-CRP: high-sensitive C-reactive protein; GDF15: Growth/differentiation factor 15; CV: cardiovascular; HF: heart failure.

**Supplementary Table S4: Multi-biomarker strategies to stratify the prognosis of patients with acute HF**

| **Author, year, ref** | **Patients (n)** | **Males (%)** | **Age (y)** | **LVEF (%)** | **Study design** | **Biomarkers** | **Measurements** | **Outcomes** |
| --- | --- | --- | --- | --- | --- | --- | --- | --- |
| Ishii  (2002) (3) | 98 | 52 | 69 | 42 | Single-center, cohort study | 1, 2, 3 | Admission | 18-m death |
| Van Kimm  (2006) (4) | 209 | 51 | 73 | 47 | Clinical trial | 4, 5, 6 | Admission | 2-m death |
| Januzzi  (2007) (5) | 208 | 51 | 61 | N/A | Clinical trial | 6, 7, 8 | Admission | 1-year death |
| Rehman  (2008) (6) | 346 | 68 | 73 | 45 | Clinical trial | 3, 6, 7, 8 | Admission | 1-year death |
| Manzano  (2009) (7) | 138 | 51 | 75 | 49 | Single-center, cohort study | 2, 6, 9 | Admission | 6-m MACE |
| Nizeki  (2009) (8) | 154 | 40 | 71 | 42 | Single-center, cohort study | 3, 10, 11, 12, 13, 14, 15 | Admission | 18-m cardiac death, 18-m HF admission |
| Zairis  (2010) (9) | 577 | 68 | 74 | 23; only pts with LVEF<35% | Multi-centre, cohort study | 3, 8, 16 | Admission | 1-m death |
| Manzano  (2011) (10) | 447 | 65 | 73 | 46; LVEF≥50% = 44% | Clinical trial | 2, 6, 7, 8 | Admission | 1-y death |
| Pascual-Figal  (2011) (11) | 107 | 56 | 72 | 47; LVEF≥50% = 51% | Single-center, cohort study | 6, 7, 17 | Admission | ~2-y death |
| Shah  (2012) (12) | 180 | 51 | 73 | N/A | Clinical trial | 5, 6, 18, 19 | Admission | 1-y death  4-y death |
| Lok  (2013) (13) | 209 | 73 | 71 | 31 | Clinical trial | 5, 6, 8, 17, 20 | Discharge | 10-y death |
| Bjurman  (2013) (14) | 131 | 53 | 73 | 43 | Single-center, cohort study | 6, 9, 17 | Discharge | 3-y death |
| Richter  (2013) (15) | 349 | 66 | 75 | LVEF≤40% = 93.9% | Multi-center, cohort study | 6, 20-31 | Discharge | 5-y death |
| Lassus  (2013) (16) | 441 | 57 | 75 | 40 | Multi-center, cohort study | 1, 2, 3, 6, 7, 15, 18, 19 | Admission | 1-m death  12-m death |
| Srinivas  (2014) (17) | 52 | 56 | 57 | 37; LVEF≥50% = 15.4% | Single-center, cohort study | 6, 8, 10, 17 | Admission, 48 h | 6-m MACE |
| Demissei  (2016) (18) | 1391 | 66 | 71 | 30; LVEF<40% = 36% | Clinical trial | 1, 5, 6, 8, 10-13, 15, 19-22, 32-66 | Sequentially | 1 & 6-m death  1-m MACE |
| Herrero-Puente  (2017) (19) | 547 | 44 | 80 | 50; *Echo data only 43% | Multi-center, cohort study | 1, 6, 19, 64, 67 | Admission | 7 &14 d death,  1-m, 3-m & 6-m death |
| Tromp  (2017) (20) | 460 | 63 | 71 | 33; LVEF≥45% = 21% | Clinical trial | 1, 7, 8, 18, 20-22, 45-48, 50-53, 55-64, 67-71 | Discharge | 18-m MACE |
| Demissei  (2017) (21) | 1653 | 68 | 70 | 30; LVEF<40% = 36% | Clinical trial | 1, 5, 6, 8, 10-13, 15, 19-22, 32-64, 66 | Sequentially | 1-m MACE  3-m death |
| Demissei  (2017) (22) | 1161 | 62 | 72 | 38; LVEF≥40% = 45.2% | Clinical trial | 5, 6, 7, 8, 9, 17, 20 | Sequentially | 6-m CV death |

List of abbreviations: 1: cTnI; 2: cTnT; 3: BNP; 4: apelin; 5: GAL-3; 6: NT-proBNP; 7: ST2; 8: hs-CRP; 9: Cys-C; 10: uric acid, 11: sodium; 12: haemoglobin; 13: creatinine; 14: creatinine clearance; 15: CRP; 16: hs-cTnI; 17: hs-cTnT; 18: MR-proANP; 19: MR-proADM; 20: GDF-15; 21: hsTNF-α, 22: myeloperoxidase; 23: MCP-1, 24: CX3CL1; 25: M-CSF; 26: hsG-CSF; 27: HGF; 28: PEDF; 29: sFAS; 30: sTRAIL; 31: sTWEAK; 32: Albumin; 33: alanine transaminase; 34: aspartate transaminase; 35: bicarbonate; 36: blood urea nitrogen; 37: chloride; 38: glucose, 39: platelet count; 40: potassium; 41: red blood cell count, 42: total cholesterol; 43: triglycerides, 44: white blood cell; 45: NGAL; 46: red angiogenin; 47: D-dimer; 48: endothelial cell-selective adhesion; 49: lymphotoxin beta receptor; 50: mesothelin; 51: neuropilin; 52: NT-proCNP; 53: osteopontin; 54: procalcitonin; 55: pentraxin-3 56: periostin; 57: polymeric immunoglobulin receptor; 58: prosaposin B; 59: receptor for advanced glycation end-products, 60: syndecan-1; 61: vascular endothelial growth receptor 1; 62: WAP four-disulphide core domain protein HE4; 63: endothelin-1; 64: interleukin-6; 65: Kidney Injury Molecule 1; 66: Troy; 67: copeptin; 68: tumor necrosis factor–associated receptor 1 a, 69: transforming growth factor b; 70: erythropoietin a; 71: estimated glomerular filtration rate.
